# Spatial and temporal evolution of urban resilience in China and analysis of barriers: Based on a sustainable development perspective

**DOI:** 10.1371/journal.pone.0285113

**Published:** 2024-02-06

**Authors:** Fengge Yao, Lin Li, Jiayuan Liang

**Affiliations:** School of Finance, Harbin University of Commerce, Harbin, China; Chongqing University, CHINA

## Abstract

With the increasing uncertainty of urban security, urban resilience construction with risk awareness and bottom-line thinking has become essential for promoting sustainable urban development. This paper measures China’s urban resilience development index (CRDI) based on 284 cities in China (except Tibet) using the entropy method from four dimensions: economic, social, environmental, and infrastructure, and analyzes it by combining coupling coordination degree and barrier factor analysis. We find that: (1) At the national level, CRDI and its sub-dimensions show an increasing trend in time, a decreasing spatial layout from coastal to inland, and a “high-high-low-low” clustering feature in space. (2) At the regional level, the CRDI is from high to low in the east, middle, and west order. The sub-dimensions are from high to low in the order of east, middle, and west for economic, social, and infrastructure resilience and from high to low in the order of east, west, and middle for environmental resilience. (3) To coupling coordination degree, the CRDI index coupling coordination is increasing in time trend but is still on the verge of dissonance. (4) Social resilience is the main obstacle factor. In the indicator layer, human resources, innovation, education, security, living, and environmental protection are the areas where CRDI coordinated development is the key to improvement. Based on the above empirical evidence, this paper proposes countermeasures to optimize urban resilience construction from four perspectives: economic, social, environmental, and infrastructure.

## 1 Introduction

In 1987, the World Commission on Environment and Development (WCED) published the report Our Common Future, defining sustainable development as development that meets the needs of the present without jeopardizing the ability of future generations to meet their needs. Implementing sustainable development strategies is of great importance for international development, which can promote the unity of ecological, economic, and social benefits and harmonize economic development with population, resources, and the environment [1]. In September 2015, world leaders adopted the "2030 Agenda for Sustainable Development" at a historic UN summit. On January 1, 2016, the 17 Sustainable Development Goals (SDGs) of the 2030 Agenda for Sustainable Development came into force. It signifies that sustainable development is the inevitable trend of future development.

As the development of various regional areas becomes closer, the intensity and scope of damage caused by disasters, accidents, and other problems are expanding [2]. In this context, ICLEI first introduced urban resilience into urban and disaster prevention research to strengthen international bottom-line thinking, improve resilience to losses, and reduce unnecessary losses in 2002 [3]. Since then, the emphasis on building resilient cities has increased in all regions [4]. For example, in 2012, UNISDR launched the Asian Cities Resilience Network for Climate Change. Furthermore, in 2013, the Rockefeller Foundation created the “Global 100 Resilient Cities” project, attracting a sizeable social response. “Ecology and resilience of cities” is one of the core elements of the new urban agenda. The current UN-Habitat pointed out that urban resilience is a new idea for sustainable urban development [5], which shows that urban resilience becomes an essential indicator for measuring the future development of the world’s cities [6, 7].

Natural disasters, accidents and disasters, and public health events pose a significant threat and unrecoverable trauma to society. A landslide occurred in Linfen City, Shanxi Province, in 2019, causing direct economic losses of over 21 million yuan. The coronavirus disease (COVID-19) outbreak in 2020 had a massive impact on China [8]. Therefore, establishing resilient cities with strong risk resistance and resilience has become imperative for urban development [9]. In October 2020, resilient cities were first proposed at the national level in China. The 14th Five-Year Plan emphasizes that urban resilience construction is as important as urban development and construction. China’s exploration to enhance urban resilience can improve the ability of Chinese cities to resist risk and provide valuable empirical evidence for urban resilience construction in the world.

This paper analyzes the urban resilience development level constructed systematically with a sample of 284 cities in China from 2011 to 2019. We propose corresponding countermeasure suggestions for strengthening urban resilience construction in the future based on the research findings. This study is divided into five main sections. The second part introduces the relevant literature. The third section describes the required analytical methods for this paper and the data required for urban resilience. The fourth part describes the spatial and temporal characteristics of urban resilience development in China at national and regional levels. Based on the empirical results, this paper puts forward targeted suggestions to improve urban resilience development in the fifth part.

## 2 Literature review

The word “resilience” is a Latin term that refers to the ability of an object to return to its original state after being deformed by an external force. The Canadian ecologist Holling (1973) first applied the concept of resilience to the field of systems ecology and defined resilience as “the ability of an ecosystem to absorb change, sustain and restore equilibrium after a transient shock” [10]. According to the definition of the Local Governments for Sustainability (ICLEI), urban resilience is the ability of cities to withstand disasters, mitigate losses, rationalize resource deployment, and recover quickly. Edward et al. (2012) consider urban economic resilience as the ability of a city to recover to its original level of development after being exposed to external shocks [11]. Moreover, Wink Rüdiger (2014) considers resilience as the ability to avoid, resist, adapt, or cope with external shocks [12]. The Urban Resilience Development Index, published in collaboration with Arup & Rockefeller foundation from 2013 to 2018, defines urban resilience as the ability of a city to survive, adapt, and thrive when subjected to any sustained chronic stress or sudden disaster shock [13].

Currently, there is a lively debate among scholars on sustainable development. Both internal and external aspects of sustainable development are included. The first internality focuses on constructing a sustainable development indicator system [14] and coordinating the various areas of sustainable development [15]. As scholars continue to study sustainable development, urban resilience is increasingly recognized as key to managing the risks and challenges emerging from global change con achieving sustainable development [16, 17]. Resilience building in different regions has a profound impact on regional development, most directly by improving regional resilience [18], indirectly by improving urban air quality, conserving resources [19], protecting ecosystems [20], promoting urban transformation [21–23], and promoting circular economy [24]. With the improvement of people’s education and health [25] and digital platforms [26], urban resilience will also increase.

As a core component of sustainable development [27], measuring resilience indicators that visually reflect urban resilience has become a hot topic of international debate. In the study of urban resilience proposition, scholars have focused more on measuring urban resilience levels.

Regarding index construction, with the development of the times and the deepening of the concept of urban resilience, it is a general trend and consensus to emphasize the systematic comprehensiveness and diversity of urban resilience elements. Among all system frameworks, the initial one is the one that contains economic, social, infrastructural, and institutional [28]. As research has progressed, the inclusion of ecological resilience has become a common way to construct an urban resilience framework. For example, Cutter Susan et al. (2008) used this framework to assess urban resilience in the United States [29], and Joerin Jonas et al. (2014) used this framework to assess urban resilience to climate hazards in Chennai, India [30]. Mcdermott Tom et al. (2016) also assessed the level of urban resilience to climate hazards in Indonesia based on this framework [31]. Xufang Mu et al. (2022) measured the urban resilience of BTH urban agglomerations from 2000 to 2018 under this framework [32]. Scholars have since started to switch perspectives or expand the framework to study urban humanity in more depth. For example, Xie Xinlu and Zheng Yan (2017) added technology and risk management to construct a climate resilience indicator system in Beijing [33], and Hudec et al. (2017) added community management capacity to analyze the differences in resilience levels between different cities in Slovakia [34]. Marjolein Spaans and Bas Waterhout (2016) assessed urban resilience in Rotterdam regarding individuals, communities, institutions, businesses, and systems [35]. Schlör Holger et al. (2017) constructed the Food, Energy, and Water (FEW) systems to compare the resilience of global and local cities [36]. Wu ChenFa et al. (2021) assessed regional flood resilience by constructing vulnerability indicators that include population characteristics, infrastructure, and land use patterns [37].

Regarding indicator measurement, the main approaches scholars use are as follows. Case study approach, Borie Maud et al. (2019) used Nairobi and Cape Town as examples to point out that lessons learned from local experiences and marginalized people are more helpful in opening up spaces for enhancing resilience [38]. Yani Wang et al. (2019) used Deqing County as an example to assess cities’ resilience, noting that their resilience is maintained at a low level [39]. Model analysis approach. Owrangi Amin et al. (2015) assessed the resilience of participating cities to climate change caused by sea level rise and river flooding through a system dynamics simulation model [40], and Feofilovs, Maksims & Romagnoli, F. (2021) validated this approach for different urban resilience scenarios assessment. Iturriza Marta et al. (2019) proposed a model that includes a resilience maturity model and a model called urban resilience dynamics to enable decision-makers to study and fully operationalize urban resilience [7]. In addition, scholars have used TOPSIS [41], geographic design [42], principal component analysis [43], case studies [44], and structural equations [45] to evaluate the level of urban resilience.

Three shortcomings can be seen in the above studies: First, in terms of research samples, from the research samples of scholars and the sources of articles, because the proposition of urban resilience was proposed earlier in foreign countries, the research results are concentrated in foreign fields, and the attention to China is low. Second, in terms of research ideas, although scholars in urban resilience measurement emphasize the general importance of systematic thinking, this thinking must be reflected in the study of urban resilience in China. Third, in terms of research method, most existing studies focus on a particular city to analyze the description of urban resilience or describe it from a global perspective, to need more analysis of spatial and temporal changes and regional heterogeneity.

In summary, this paper has three major innovations. First, this paper analyzes the development of urban resilience in China with a research sample of 284 cities in China from 2011 to 2019. Second, from a systematic and multidimensional perspective, this paper constructs an urban resilience index from four dimensions: economic, social, environmental, and infrastructure, and analyzes and evaluates it. Third, this paper analyzes urban resilience in terms of global and regional heterogeneity and innovatively introduces coupling coordination analysis and barrier factor analysis to analyze and study the internal development of the urban resilience system. In addition, it is worth mentioning that this paper’s research dimensions and framework apply to China and have specific guiding significance for developing urban resilience in other countries.

## 3 Methodology and data

### 3.1 Research methodology

#### 3.1.1 Entropy value method

In this paper, the entropy method is used to measure urban resilience. German physicist Clausius founded the earliest entropy theory in 1856 [46]. As a physics concept, entropy refers to a measure of the degree of chaos in a system. In 1971, George Escue Logan published his influential book The Entropy Law and the Economic Process, which was the first to apply "entropy" to economic analysis [47]. In 1980, in Jeremy Rifkin and Ted Howard’s book Entropy: A New World View, the application of the concept of entropy in economics and the challenges facing Western capitalist societies were further emphasized through the lens of entropy [48]. In its application, the entropy method objectively reflects the weight of each indicator by the weighted value determined by correlating the original data of each indicator, which has the advantage of avoiding the influence and bias of subjective factors to a certain extent [49, 50] and is therefore widely used in economics.

Suppose that the urban resilience index of m cities for n years is to be measured, and the number of required indicators is z. Then x_ijt_ is the j indicator of the city in a year t. Based on the setting, the steps of CRDI measurement are as follows.

In the first step, the extreme value method standardizes the data based on the indexes’ directivity. While avoiding zero values in the calculation of the indexes, this paper non-zeroes the indexes based on standardization. If the directivity of the index is positive, it is shown in Eq (1), and if the directivity of the index is negative, it is shown in Eq (2).


$${x_{ijt}}^{\prime }=[\left(x_{ijt}-minx_{ijt}\right)/\left(maxx_{ijt}-minx_{ijt}\right)]\times 0.99+0.1$$ (1)

$${x_{ijt}}^{\prime }=\left[(maxx_{ijt}-x_{ijt}\right)/\left(maxx_{ijt}-minx_{ijt}\right)]\times 0.99+0.1$$ (2)

In the second step, the indicators are normalized as shown in Eq (3).


$$Y_{ijt}=x_{ijt}/\sum_{t=1}^{m}\sum_{i=1}^{n}x_{ijt}$$ (3)

In the third step, the entropy value of each indicator (*e*_*j*_) is calculated as shown in Eq (4). Where *k* = 1/ln (*mn*)

$$e_{j}=-k\sum_{t=1}^{m}\sum_{i=1}^{n}Y_{ijt}ln(Y_{ijt})$$ (4)

In the fourth step, as shown in Eq (5), the information entropy of each indicator (*d*_*j*_) is calculated based on the entropy value. The greater the information entropy, the greater the utility of the indicator for the measure.


$$d_{j}=1-e_{j}$$ (5)

In the fifth step, based on the information entropy, the weight of each subindex is calculated, as shown in Eq (6). At this point, the evaluation formula of each dimension of urban resilience is shown in Eq (7). Where w_j_ is the resilience index for each dimension and *CRDI*_*a*_ is the composite city resilience index.


$$w_{j}=d_{j}/\sum_{j=1}^{z}d_{j}$$ (6)

$${CRDI}_{a}=\sum_{j=1}^{z}w_{j}{x_{ijt}}^{\prime }$$ (7)

#### 3.1.2 Spatial spillover effect

In order to observe the spatial distribution of CRDI, this paper uses the global Moran index statistic for spatial autocorrelation analysis to measure the strength of spatial association among provinces. The Moran index is calculated as shown in Eq (8). Where n is the number of samples, ω_ij_ is the spatial weight matrix. x and $\bar{x}$ are the variables and their mean values, respectively.


$$Moran‘‘I=\frac{n\sum_{i=1}^{n}\sum_{i\neq j}^{n}\omega_{ij}(x_{i}-\bar{x})(x_{j}-\bar{x})}{\sum_{i=1}^{n}\sum_{i\neq j}^{n}\omega_{ij}\sum_{i=1}^{n}{\left(x_{i}-\bar{x}\right)}^{2}}$$ (8)

#### 3.1.3 Coupling coordination degree

In order to further study the development status of coupling and coordination among the internal indicators of CRDI and the development level [38], this paper adopts a coupling model to analyze coupling model to judge the degree of role among the internal indicators of CRDI in China.

The four indices are first dimensionless processed, consistent with the formulas of Eqs (1) and (2) in the entropy weighting method. After dimensionless processing, the coupling degree of the internal indices of CRDI is calculated. In the coupling model, the coupling degree reflects the interaction relationship between the variables within the subsystem. This paper examines the mutual coordination status among CRDI subsystems and studies the interaction among the CRDI subsystem indices in different regions of China. The formula of the coupling degree is shown in Eq (9). C is the coupling degree index, a, b, c, and d are the dimensionless ECO, SCO, ENV, and INF.


$$C={\left\{\frac{a\times b\times c\times d}{{\left[(a+b+c+d)/4\right]}^{4}}\right\}}^{1/4}$$ (9)

There are two kinds of coordination index algorithms: entropy and equal weights. This paper uses the entropy weight method to present the coordination index with the formula shown in Eq (10), where the weight corresponds to the dimension. In this paper, the final calculation results are presented in Table 2. *T*_1_ is the coordination index, *w*_*a*_, *w*_*b*_, *w*_*c*_ and *w*_*d*_ are the weights of ECO, SCO, ENV, and INF, respectively.


$$T_{1}=a\times w_{a}+b\times w_{b}+c\times w_{c}+d\times w_{d}$$ (10)

Based on the above coupling degree and the coordination index, the coupling coordination index between CRDI subsystems is calculated, its formula is shown in Eq (11), and its coupling coordination index level is shown in the table. According to the model, the value range of the coupling coordination degree is mainly between 0 and 1. When the value is closer to 1, the coupling coordination degree of CRDI subsystems is better. Based on this, the coupling coordination degree index between CRDI subsystems is shown in Table 1. *D*_2_ is the coupling coordination degree.


$$D_{2}={\left(C\times T_{1}\right)}^{1/2}$$ (11)

**Table 1:** Distribution of coupling coordination degree index levels.

Coupling	Coordination	D-value interval	Degree of coupling coordination
Degree	Degree		
Extremely	Low	(0.0~0.1)	Extremely dysfunctional
Uncoupled	coordination	[0.1~0.2)	Severe dissonance
Moderately	Moderate	[0.2~0.3)	Moderate disorder
coupled	coordination	[0.3~0.4)	Mild disorder
		[0.4~0.5)	Nearly out of tune
Basic	Basic	[0.5~0.6)	Barely coordinated
coupling	coordination	[0.6~0.7)	Primary coordination
		[0.7~0.8)	Moderate extreme coordination
Highly	Extremely	[0.8~0.9)	Good Coordination
coupled	coordinated	[0.9~1.0)	High Quality Coordination

#### 3.1.4 Barrier factor analysis

Calculating the barrier degree is as follows. First the factor contribution degree F is calculated, which is shown in Eq (12). The criterion level weight is set to W, and the indicator level weight is set to P. Second, the indicators are standardized. This step is consistent with the standardization step in the entropy weight method. In the third step, the barrier degree I of each factor is calculated, which is shown in Eq (13). The formula for calculating the barrier degree of its indicator layer is shown in Eq (14). Where *j* represents the indicator serial number and m represents the total number of indicators. The barrier degree formic acid method of its criterion layer is shown in Eq (15).


F=W×P (12)

$$I=1-{x_{ijt}}^{\prime }$$ (13)

$$o_{m}=\frac{F\times I}{\sum_{m=1}^{n}\left(F\times I\right)}$$ (14)

$$U=\sum o_{m}$$ (15)

### 3.2 Data and descriptive statistics

#### 3.2.1 Theoretical foundation

Resilient cities are a frontier topic in urban and environmental research. In this research field, sustainable development theory, risk society theory, and resilience theory can be used as the theoretical basis for the analysis of resilient cities, and this paper constructs an urban resilience system based on this basis in conjunction with the Disaster Resilience of Place (DROP) framework model.

1. Theory of sustainable development. The theory of sustainable development refers to development that meets the needs of the present without posing a danger to the ability of future generations to meet their needs, with equity, sustainability, and commonality as the three basic principles, and its ultimate goal is to achieve joint, coordinated, equitable, efficient and multidimensional development. In order to better achieve sustainable development, 17 Sustainable Development Goals (SDGs) were officially adopted at the UN Sustainable Development Summit in 2016, aiming to shift to a sustainable development path by thoroughly addressing the social, economic, and environmental dimensions of development in an integrated manner between 2016 and 2030.Risk society theory. Beck, U. (1999) proposed the risk society theory, pointing out that with the advent of risk society, risks in economic, political, cultural, ecological, and energy aspects will also increase; not only regional risks coexist with global risks [51]. These risks are often intertwined and interpenetrated, and are increasingly difficult to predict and prevent. Therefore, it is very necessary for cities to enhance their ability to face risks.
2. Resilience theory. Resilience theory originated from ecology and is widely used in the research fields of climate change adaptation, sustainable cities, resilient cities, etc. The resilience paradigm better reflects the multi-stability, self-adaptive, and dynamic change characteristics of social-ecological complex systems than risk analysis. The concept of resilience emphasizes transforming risks into opportunities through proactive and forward-looking adaptation to drive change, innovation, and transformation.
3. The Disaster Resilience at the Local Level (DROP) framework model The DROP framework is built on the interdependencies between the components of urban systems, combined with systems thinking. The model is used to study natural disasters and other emergencies, so the model applies to the study in this paper. This paper uses the DROP model as the basic logic to analyze urban disaster resilience systematically. Although the DROP model focuses more on the social dimension, the economy, environment, and foundation are interlinked. Meanwhile, in existing studies, cities are considered a complex system that includes different components such as social, economic, infrastructural, natural, interaction, and interdependence [52].

In summary, sustainable development theory requires us to promote orderly development in all fields in a comprehensive manner. Risk society theory points out that the current risk to development is complex and multidimensional, and resilience theory points out that a resilient system is a multidimensional homeostasis. Meanwhile, the DROP model reflects that the impact of emergencies usually has a cascading effect; therefore, the resilience system as an evaluation system for resisting emergencies requires us to analyze the resilience level of cities from a system perspective [53–56]. Therefore, in this paper, we construct an urban resilience framework containing economic, social, ecological, and primary energy as sub-dimensions of integration to investigate the level of resilience development in Chinese cities.

#### 3.2.2 Index selection

This paper combines the above theoretical analysis from the system analysis perspective. It uses the DROP model as the basic logic to construct an evaluation system of urban disaster resilience containing four aspects: economy, society, environment, and infrastructure, whose index measures are shown in Table 2.

**Table 2:** Description of the City Resilience Development Index (CRDI) indicator system.

Resilience theme	Weight	Elemental Indicators	Weight	Measurement Indicators	Indicators
Economic Resilience	0.13	Level of Development	0.15	GDP per capita	+
				Share of Tertiary Industry	+
				Income per capita	+
				Market Potential	+
		Manpower Level	0.21	Employment	+
				Number of students in school	+
				Population growth rate	+
				Population Density	+
		Investment Level	0.27	Fixed Capital Stock	+
				Digital Inclusive Finance	+
				Financial institution savings	+
				Foreign Direct Investment	+
				Fiscal Investment	
		Innovation level	0.37	Fiscal Science and Technology Spending	+
				Technology Employment Level	+
				Innovation and Entrepreneurship Index	+
				Proportion of Private Economy	
Social Resilience	0.17	Social Medical	0.11	Number of Hospitals	+
				Number of Beds	+
				Number of physicians per 10,000 people	+
				Number of health workers per 10,000 people	+
		Social Education	0.33	Number of undergraduate and specialist teachers per 10,000 people	+
				Number of undergraduate and specialized schools	+
				Number of elementary school students	+
				Financial Education Expenditure	+
		Social Security	0.32	Number of basic pension insurance participants per 10,000 people	+
				Number of basic medical insurance participants per 10,000 people	+
				Number of unemployment insurance participants per 10,000 people	+
				Number of people employed in residential services per 10,000 people	+
		Social Life	0.24	Cell phone subscribers	+
				Internet users	+
				Number of public transport users per capita	+
				Park construction level	+
				Number of cultural workers per 10,000 people	+
				Book collection per capita	
Environmental Resilience	0.62	Environmental Quality	0.19	Annual average concentration of PM2.5	—
				Greening rate of construction land	+
				Green space construction level	+
		Environmental Protection	0.81	Environmental regulation intensity	+
				Domestic waste treatment rate	+
				Number of environmental employees per 10,000 people	+
				Number of government environmental protection-related words and phrases	+
Basic Resources	0.09	Infrastructure	0.65	Urban road area	+
				Total postal service revenue	+
				Length of drainage pipes	+
				Municipal investment level per unit area	+
		Energy Supply	0.35	Urban water supply level	+
				Level of urban electricity supply	+
				Level of city gas supply	+
				City LPG supply level	+

#### 3.2.3 Study area

This paper selected 284 cities in China as a sample and divided them into three regions: eastern, central, and western, from the coast to inland. The eastern region mainly consists of Beijing, Tianjin, Hebei (9 cities), Liaoning (14 cities), Shanghai, Jiangsu (13 cities), Zhejiang (11 cities), Fujian (9 cities), Shandong (16 cities), Guangdong (21 cities), Guangxi (14 cities), and Hainan (2 cities), with a total of 114 cities. The central region mainly consists of Shanxi (11 cities), Inner Mongolia (9 cities), Jilin (8 cities), Heilongjiang (12 cities), Anhui (16 cities), Jiangxi (11 cities), Henan (17 cities), Hubei (12 cities), Hunan (13 cities) nine components, a total of 109 cities. The western region mainly consists of Chongqing, Sichuan (18 cities), Guizhou (4 cities), Yunnan (8 cities), Shaanxi (10 cities), Gansu (12 cities), Qinghai (1 city), Ningxia (5 cities), Xinjiang (2 cities) eight provinces, a total of 61 cities.

#### 3.2.4 Data sources

The data sources in this paper are described as follows. The economic resilience innovation and entrepreneurship index and inclusive digital finance index are from the Peking University data platform, and the rest are from China City Statistical Yearbook 2012–2020. The social and primary resilience data are obtained from the 2012–2020 China Urban Statistical Yearbook. The fixed capital stock is calculated using the perpetual inventory method with a depreciation rate of 9.6%. In environmental resilience, government word frequency data are obtained from the Chinese government website, pm2.5 data are obtained from the data of atmospheric composition analysis by Washington University, and the rest are obtained from the 2012–2020 China Urban Statistical Yearbook. Among them, environmental regulation is obtained by weighting the ratio of sulfur dioxide, soot, and nitrogen oxides to GDP and then taking the inverse of it. The interpolation method is used to fill this paper with the missing data. The Tibetan region is excluded from this paper because of the problem based on the data availability. All price-related variables in this paper are deflated, with 2011 as the base period.

## 4 Analysis of measurement results

### 4.1 Spatial and temporal characteristics of CRDI at the national level

#### 4.1.1 CRDI national-level temporal characteristics

**Fig 1:**
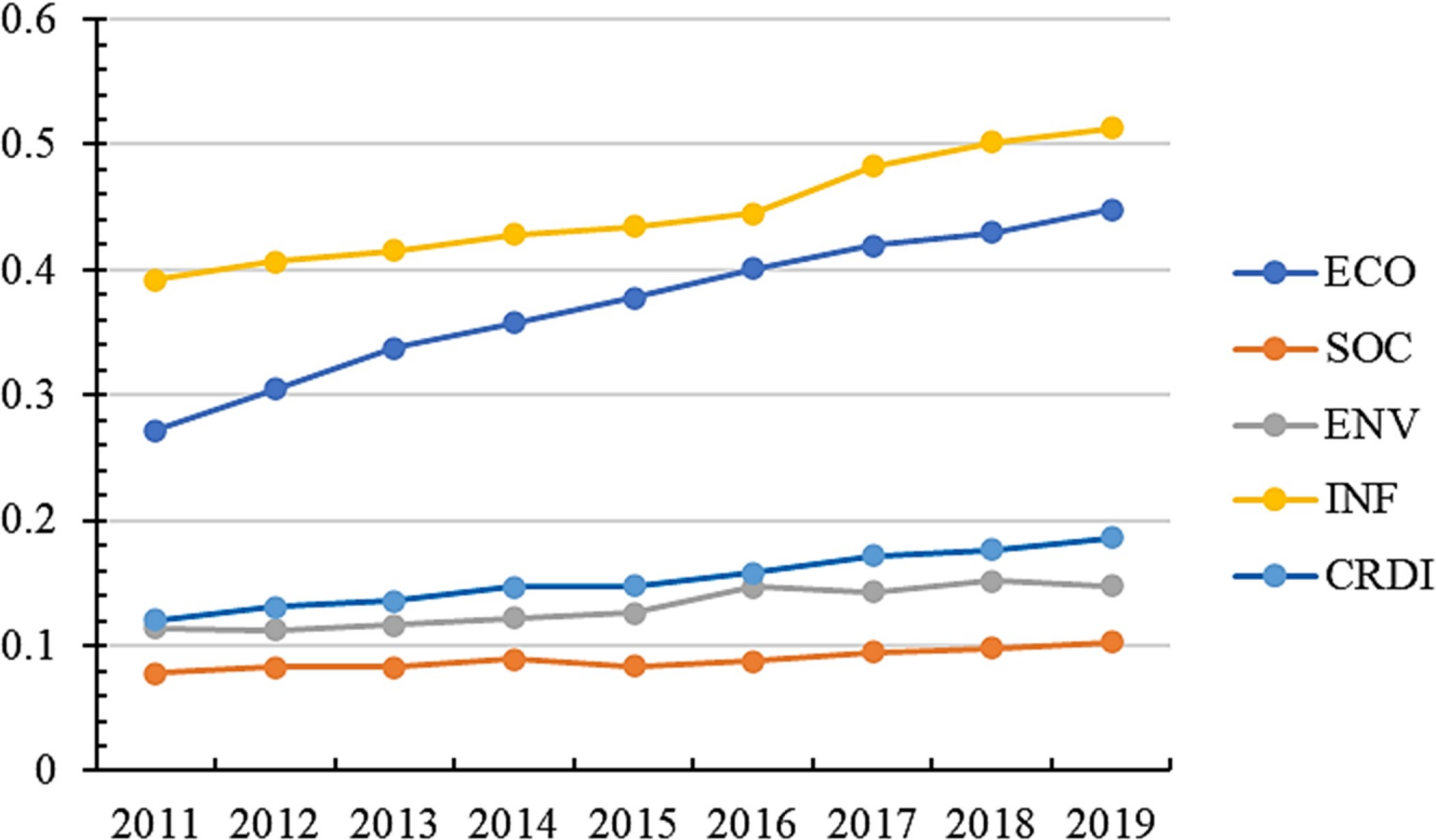
Trend of total CRDI index and sub-dimensions (median).

According to the calculated statistics, the mean value of China’s urban development resilience level is only 0.193, and the median value is 0.153. Only 87 cities exceed the mean value, accounting for 31% of the total sample, showing that the current level of China’s urban resilience is low and significant regional differences exist. When the data differ, the median can better reflect the general level than the mean value. This paper uses the median to describe the changing trend of the total CRDI index and sub-dimensions. Their trend change is shown in Fig 1.

China’s CRDI increased from 0.120 in 2011 to 0.186 in 2019. The central government is paying more attention to urban construction as the national concept of five-in-one construction is deepening. In terms of sub-dimensions, there is a general lack of internal balance in the construction of the horizontal system of urban resilience in China. From 2011 to 2019, each dimension of CRDI showed an upward trend, with the level of development foundation from high to low in the order of essential resilience (0.392), ECO (0.272), environmental resilience (0.114), and SCO (0.077), while the level of development from high to low in the order of ECO (0.176), essential resilience (0.121), and SCO (0.033), and ENV (0.025). SCO is the short area for developing the urban resilience system. Among CRDI, ENV showed an upward trend from 2011 to 2016, while the development trend became stable after 2016. With the increasing awareness of environmental protection in China, many environmental regulation and environmental credit policies were introduced between 2010 and 2013, such as the emissions trading policy proposed in 2007, the low-carbon city policy proposed in 2010, and the green credit policy proposed in 2012. At the beginning of the policy proposal, regional production saw a certain magnitude of change. The region also improved ENV with the continuous promotion of regulatory policies and sustainable development. However, the regional ENV stabilized at around 0.285 after 2016, which is a low level, and it is necessary to find new ways of environmental governance.

#### 4.1.2 CRDI national-level spatial characteristics

**Fig 2:**
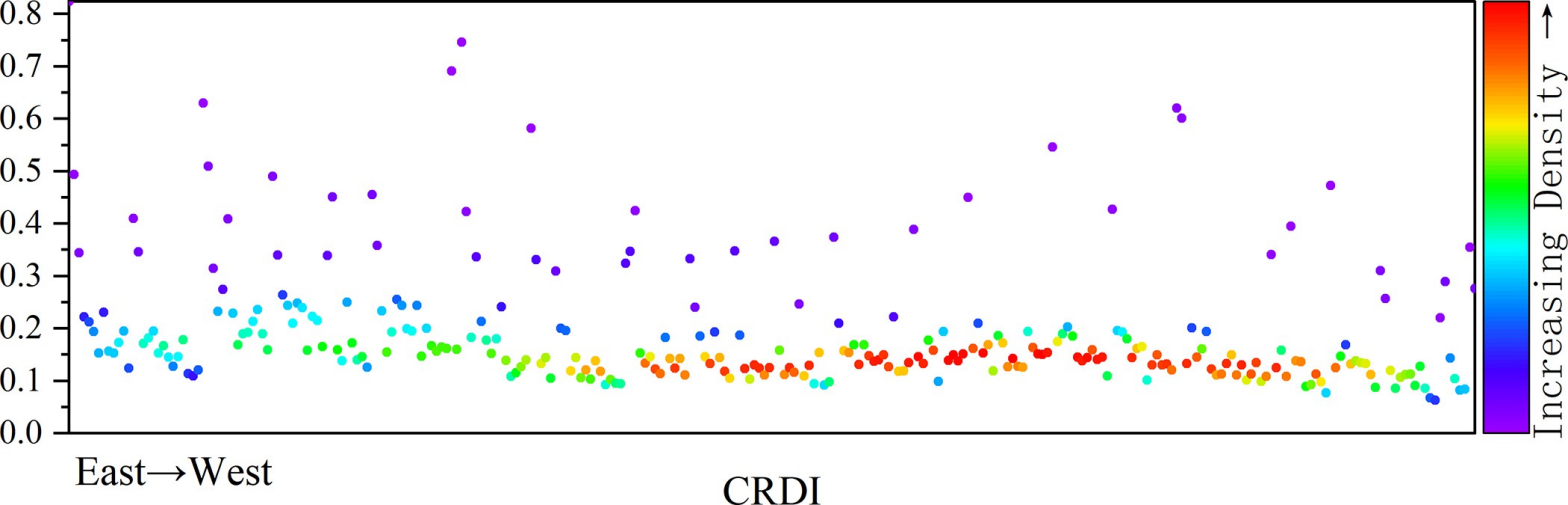
CRDI distribution map.

**Fig 3:**
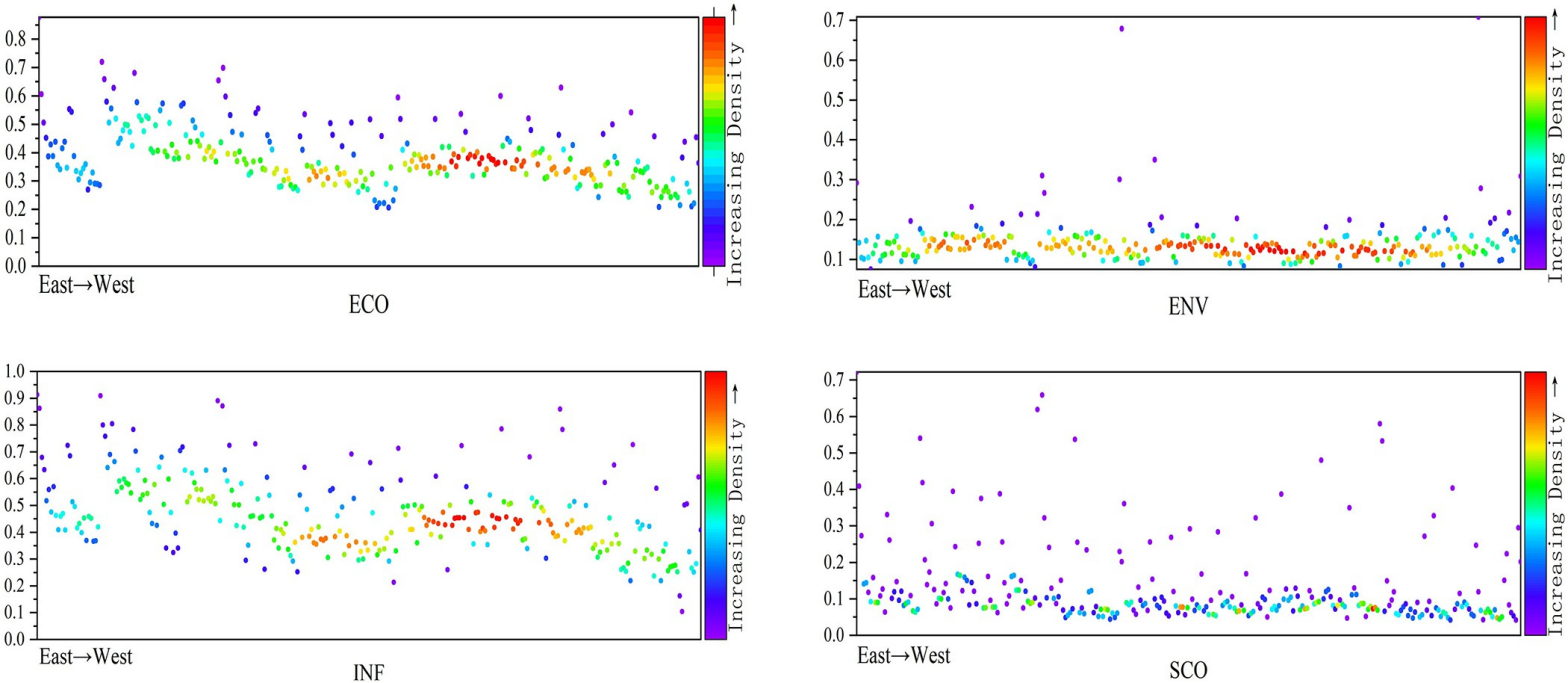
Distribution of CRDI sub-dimensions.

At the national level, China’s CRDI showed an upward trend from 2011 to 2019, particularly clear in southeastern China’s coastal zone (Fig 2). Regarding the average urban resilience from 2011 to 2019, the distribution of CRDI in China indicates a decreasing distribution pattern from coastal to inland areas. From a sub-dimensional perspective, ECO, SCO, and INF all show a distribution pattern that is more similar to CRDI (Fig 3). The decreasing distribution of ENV from coastal areas to inland areas is relatively weak. In contrast, the opposite distribution of ENV ECO exists in some cities. According to the environmental Kuznets curve, the higher the economic development, the higher the intensity of production and life, and the anthropogenic activities will cause pressure on the environment, so the environmental resilience performance of economically developed areas is not outstanding.

The calculation results in descending order of CRDI are shown in the table in S1 Table in S1 File, and Table 3 indicates the top ten and bottom ten rankings of CRDI and its sub-dimensions. Combining Tables 3 and 4, overall, among the top ten cities, Beijing, Shanghai, Chongqing, and Tianjin are Chinese municipalities, and Guangzhou and Shenzhen are the other two first-tier cities in China besides Beijing and Shanghai. As new first-tier cities, Chengdu, Dongguan, Wuhan, and Nanjing are also in this ranking. Regarding sub-dimensional rankings, cities in the top 10 perform well and rank higher in the three dimensions of ECO, SCO, and INF, while their performance in ENV is relatively poor. Most first-tier cities and municipalities are directly under the central government, and new first-tier cities are in the eastern coastal region. Their inherent location advantages can attract more talent and technologies, high population density, and high social demands. Their leading development base can receive more attention, so ECO, SCO, and INF perform outstandingly. According to the environmental Kuznets curve, the more active human activities in developed regions will cause certain pressure on ecology. China is in the critical period of carbon peak, so some CRDI-leading cities in this stage have relatively inferior performance in ENV. However, the continuous promotion of environmental regulations such as government carbon trading policies, emission trading policies, emission fees, and green finance have also optimized the environmental resilience of developed cities, resulting in an overall increase in CRDI.

**Table 3:** Top 10 and bottom 10 ranking of CRDI and its sub-dimensions.

Top 10 cities in CRDI ranking										
City	ECO	Rank	SCO	Rank	ENV	Rank	INF	Rank	CRDI	Rank
Beijing	0.877	1	0.722	1	0.292	7	0.913	1	0.824	1
Shenzhen	0.699	3	0.659	2	0.310	4	0.871	4	0.746	2
Guangzhou	0.655	6	0.619	3	0.214	12	0.891	3	0.691	3
Shanghai	0.720	2	0.540	5	0.176	30	0.909	2	0.630	4
Chongqing	0.463	57	0.580	4	0.166	37	0.860	6	0.620	5
Chengdu	0.629	7	0.533	7	0.186	23	0.784	10	0.601	6
Dongguan	0.540	24	0.537	6	0.159	48	0.730	13	0.582	7
Wuhan	0.600	10	0.480	8	0.146	88	0.786	9	0.546	8
Nanjing	0.659	5	0.419	9	0.166	38	0.800	8	0.509	9
Tianjin	0.606	9	0.409	10	0.142	101	0.863	5	0.494	10
……	……	……	……	……	……	……	……	……	……	……
Cities in the bottom ten of CRDI ranking										
City	ECO	Rank	SCO	Rank	ENV	Rank	INF	Rank	CRDI	Rank
Pingliang	0.244	272	0.051	270	0.139	112	0.274	267	0.091	275
Baoshan	0.268	260	0.052	268	0.114	215	0.261	272	0.089	276
Shangluo	0.260	268	0.053	264	0.116	206	0.237	279	0.087	277
Qingyang	0.252	269	0.048	277	0.131	150	0.252	277	0.086	278
Baiyin	0.243	273	0.043	282	0.121	187	0.329	244	0.086	279
Zhongwei	0.221	278	0.042	283	0.155	64	0.283	265	0.084	280
Guyuan	0.211	281	0.053	265	0.123	181	0.251	278	0.082	281
Lincang	0.245	270	0.042	284	0.128	163	0.219	280	0.077	282
Xiding	0.242	274	0.044	281	0.097	261	0.163	283	0.067	283
Longnan	0.215	280	0.050	273	0.099	257	0.104	284	0.063	284

**Table 4:** CRDI overall and sub-dimensional spatial effect indices.

Year	CRDI	ECO	SCO	ENV	INF
2011	0.032***	0.107***	0.019***	0.047***	0.079***
	(5.605)	(17.477)	(3.686)	(8.799)	(13.062)
2012	0.034***	0.107***	0.023***	0.045***	0.078***
	(5.993)	(17.554)	(4.255)	(8.709)	(12.94)
2013	0.034***	0.107***	0.022***	0.037***	0.087***
	(6.021)	(17.576)	(4.137)	(7.126)	(14.363)
2014	0.038***	0.108***	0.027***	0.048***	0.086***
	(6.571)	(17.683)	(4.828)	(8.606)	(14.201)
2015	0.036***	0.11***	0.024***	0.043***	0.09***
	(6.287)	(17.961)	(4.374)	(7.837)	(14.737)
2016	0.033***	0.116***	0.022***	0.038***	0.088***
	(5.892)	(18.972)	(4.021)	(7.08)	(14.502)
2017	0.035***	0.123***	0.023***	0.041***	0.088***
	(6.105)	(20.061)	(4.264)	(8.305)	(14.518)
2018	0.033***	0.126***	0.021***	0.044***	0.09***
	(5.863)	(20.558)	(3.926)	(8.497)	(14.755)
2019	0.036***	0.133***	0.023***	0.049***	0.091***
	(6.328)	(21.671)	(4.281)	(8.936)	(15.017)

Standard errors in parentheses

*** p<0.01

** p<0.05

* p<0.1

According to the first law of geography, “everything is related, and things that are close to each other are more closely related” this paper adopts the distance matrix to analyze the spatial distribution of CRDI and its sub-dimensions. It uses the statistical value of the Moran index and significant level as the basis for describing the spatial spillover effect, and the results are shown in Table 4. From the perspective of spatial layout, there is a significant positive spillover effect for the overall CRDI index and its sub-dimensions, showing that the spatial distribution of CRDI and its sub-dimensions show a “high-high-low-low” clustering feature.

Overall, the Moran index of urban resilience development in China is significant, indicating that resilient city construction can generate spillover effects. However, due to the low level of importance attached to urban resilience construction in the region, the level of resilience development varies less among regions. Therefore, the Moran index is less volatile. Regarding sub-dimensions, the spillover effects are ENV, INF, ENV, and SCO in the order from high to low.

ECO is developing rapidly, and the spillover effect on the surrounding areas shows an upward trend. During 2011–2016, ENV and ECO are roughly negatively correlated, indicating that when the spillover effect of economic development increases, the surrounding areas will take advantage of their location to absorb advanced industries from the developed surrounding areas to promote economic development, which there may be industries extruded due to environmental control, causing environmental pressure. However, after 2016, with the concept of green and sustainable development, low-carbon cities, and policies such as emission rights, regional development pays more and more attention to developing a development model in which economy and ecology coexist, so the spillover effect becomes positively correlated after 2016.

The spillover effect of INF is on the rise before 2016, and the spillover effect is stable after 2016. On the one hand, the energy supply is limited. When the intensity of energy use reaches a certain level, it will provoke people’s awareness of the crisis of future energy use, thus protecting energy use, which also has a spillover effect on neighboring regions; on the other hand, infrastructure construction is improving with regional facilities, and the gap between their development levels is also closing, thus making the spillover effect maintain a relatively stable state after 2016.

**Fig 4:**
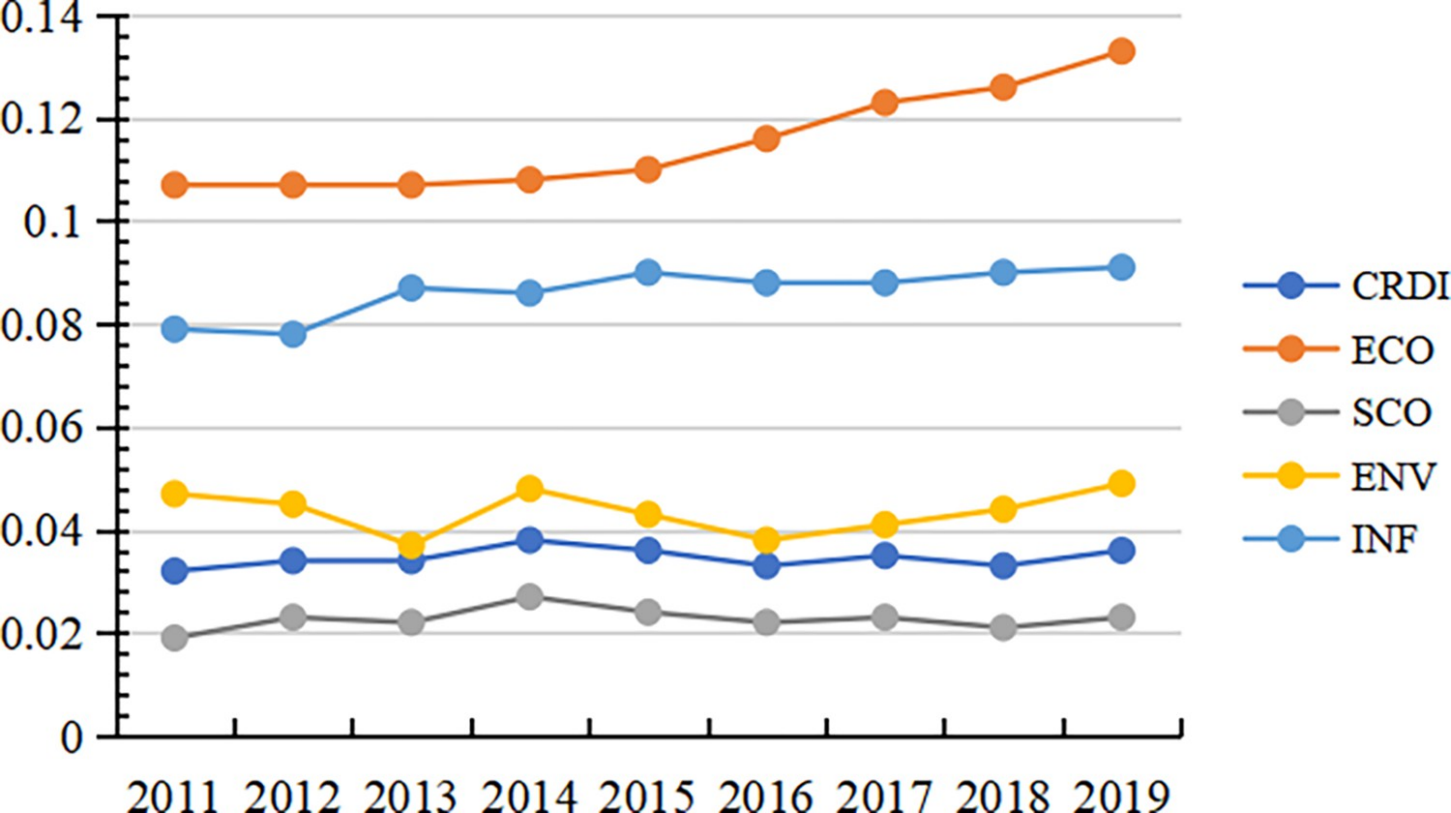
Moran index fluctuation chart.

SCO is the positive and weakest spillover effect. On the one hand, the high level of development of social infrastructure in the periphery leads to local optimization of its social services; on the other hand, social services in the periphery attract population migration to the periphery, and population migration stimulates local upgrading of its social resource construction, thus generating positive spillover effects. However, the high demand for social services and rapid changes on the demand side put pressure on the supply of social services. Conversely, regional social services sometimes need to be tailored to local conditions, leading to low spillover effects of social resilience (Fig 4).

### 4.2 Spatial and temporal characteristics of CRDI regional level

#### 4.2.1 CRDI regional time-varying characteristics

In this paper, for analysis, China is divided into three major regions according to the regional division of China the eastern, central, and western regions. Table 5 indicates the performance of the three major divisions and the national CRDI. From a static perspective, the eastern region has a higher level of resilience than the national level; the central region ranks second and has the most balanced regional development; the western region ranks third in resilience and has the most significant difference between cities.

**Table 5:** CRDI performance of the three major divisions 2011–2019.

Area	Number of Cities	Average value	Standard Deviation	Coefficient of variation	Median
National	284	0.193	0.118	0.611	0.153
East	111	0.230	0.138	0.600	0.222
Central	60	0.169	0.080	0.473	0.146
West	109	0.165	0.117	0.709	0.127

**Fig 5:**
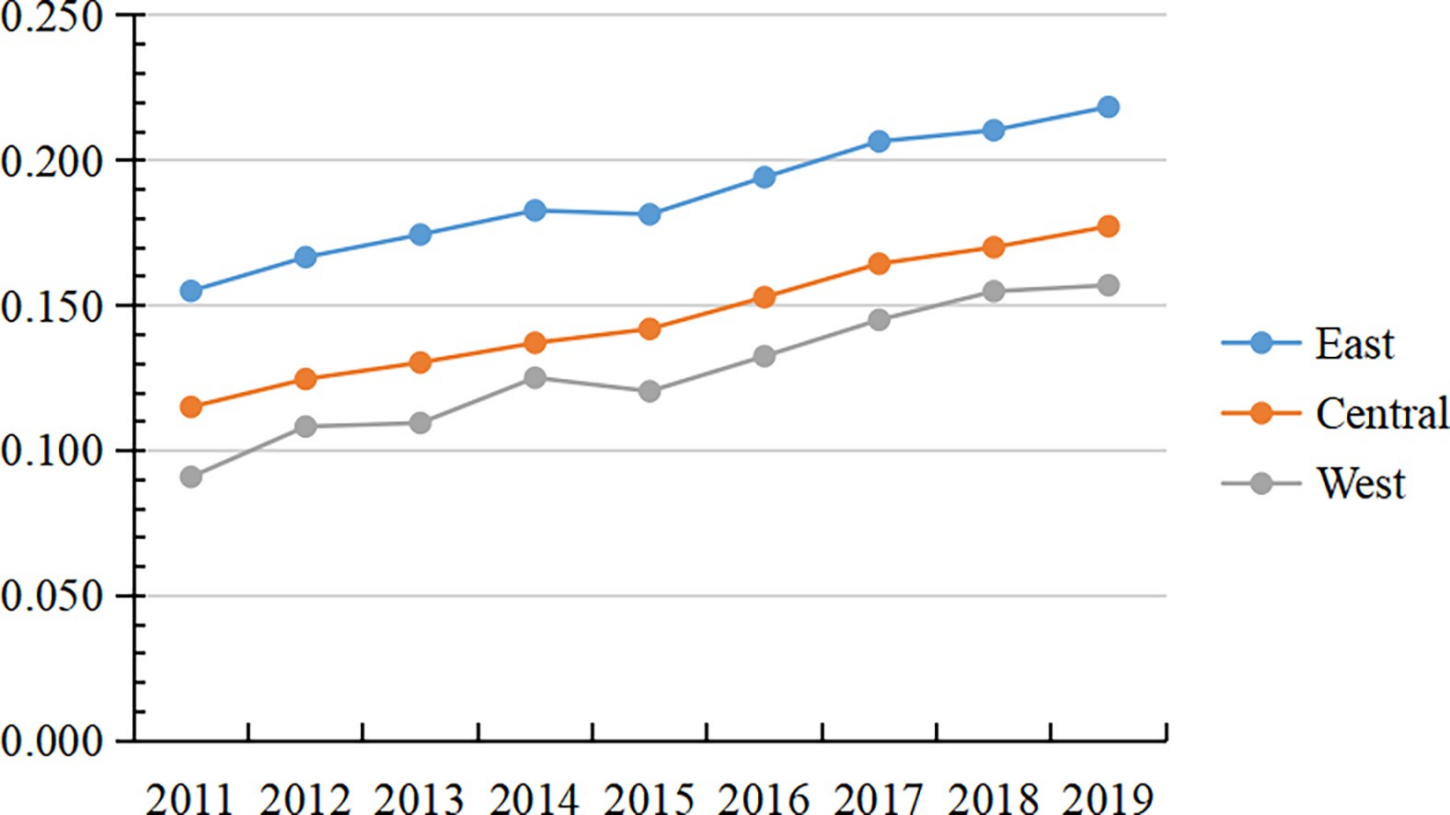
Time trends of the three major CRDI divisions.

**Fig 6:**
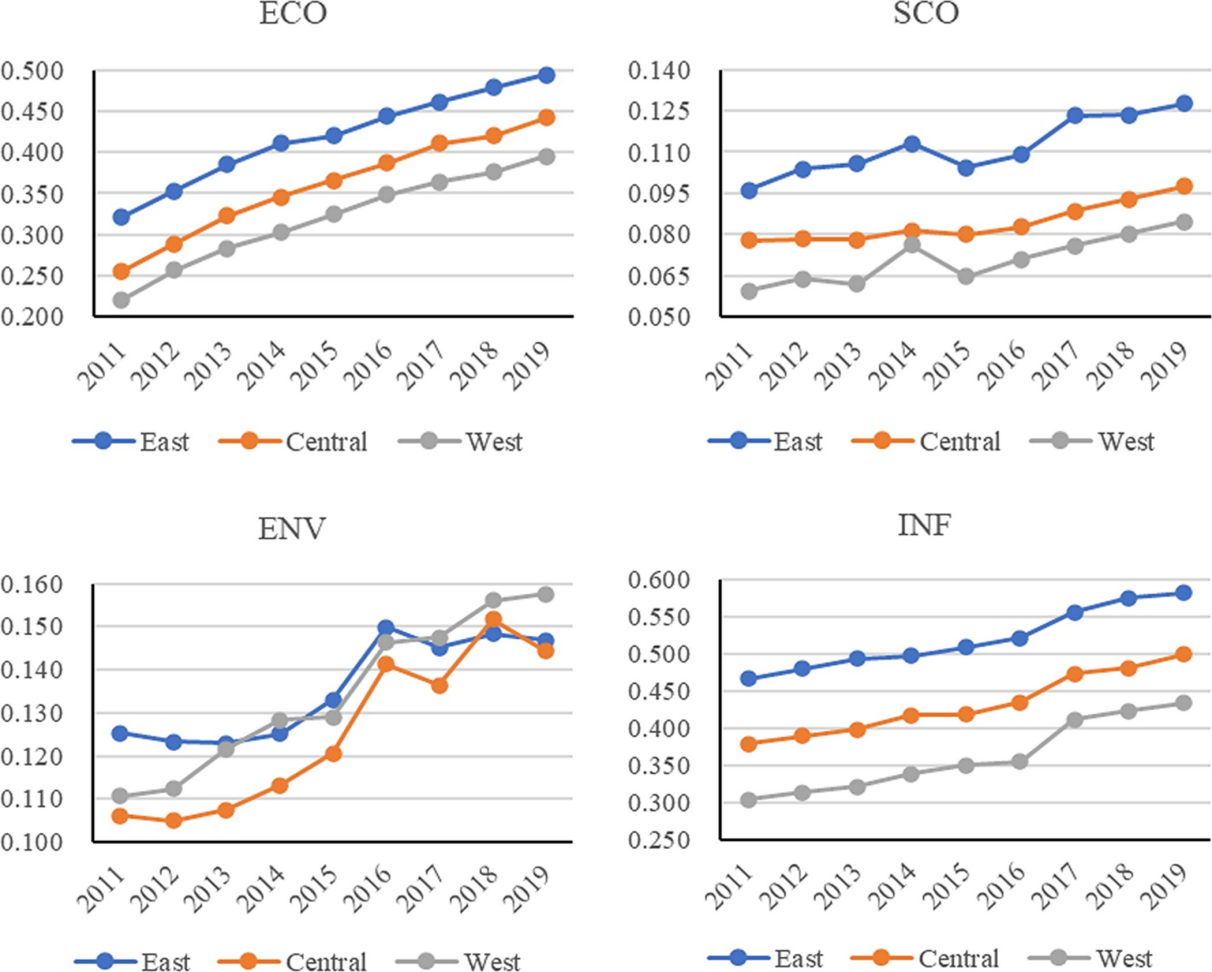
Trend of CRDI sub-dimensions in three major divisions, 2011–2019.

Fig 5 indicates the temporal development trend of CRDI in the three significant sub-regions from 2011 to 2019; from the dynamic perspective, overall CRDI indicates an upward trend, and the changing trend is the same. The eastern region has the best development, followed by the central and western regions, showing that the three significant sub-regions are strengthening the importance of urban resilience with time. However, there is regional heterogeneity because of the different regional development bases. The sub-dimensions temporal trends are shown in Fig 6. ECO, SCO, ENV, and INF in the three significant sub-districts indicate an increasing trend. ECO, SCO, and INF from high to low are eastern, central, and western. However, ENV is high to low in the eastern, western, and central order. As the government’s attention to regional development rises, the region’s ECO, SCO, and INF strengthen. Because of the location characteristics and primary innate conditions of the East, Central, and West sub-regions, there is usually a better arrangement of the east than the central and the west. The eastern region has a good development base, the industrial structure is low-pollution and high-value-added industries, and it is attractive to high-tech talents and technologies in the environmental field, which makes it leading at the environmental level. The western region has a weaker development base. It is sparsely populated, with complex terrain, less human-made, and less ecological pressure.

In contrast, the central region, with a dense population and an industrial structure dominated by the secondary industry, has a high pollution intensity and high environmental pressure. In addition, the government’s environmental regulation policies are relatively more important to the eastern region, and the pilot areas are more concentrated in the eastern region. Therefore, ENV indicates the characteristics of East, West, and Central arrangements.

#### 4.2.2 Spatial characteristics of CRDI regional level

**Fig 7:**
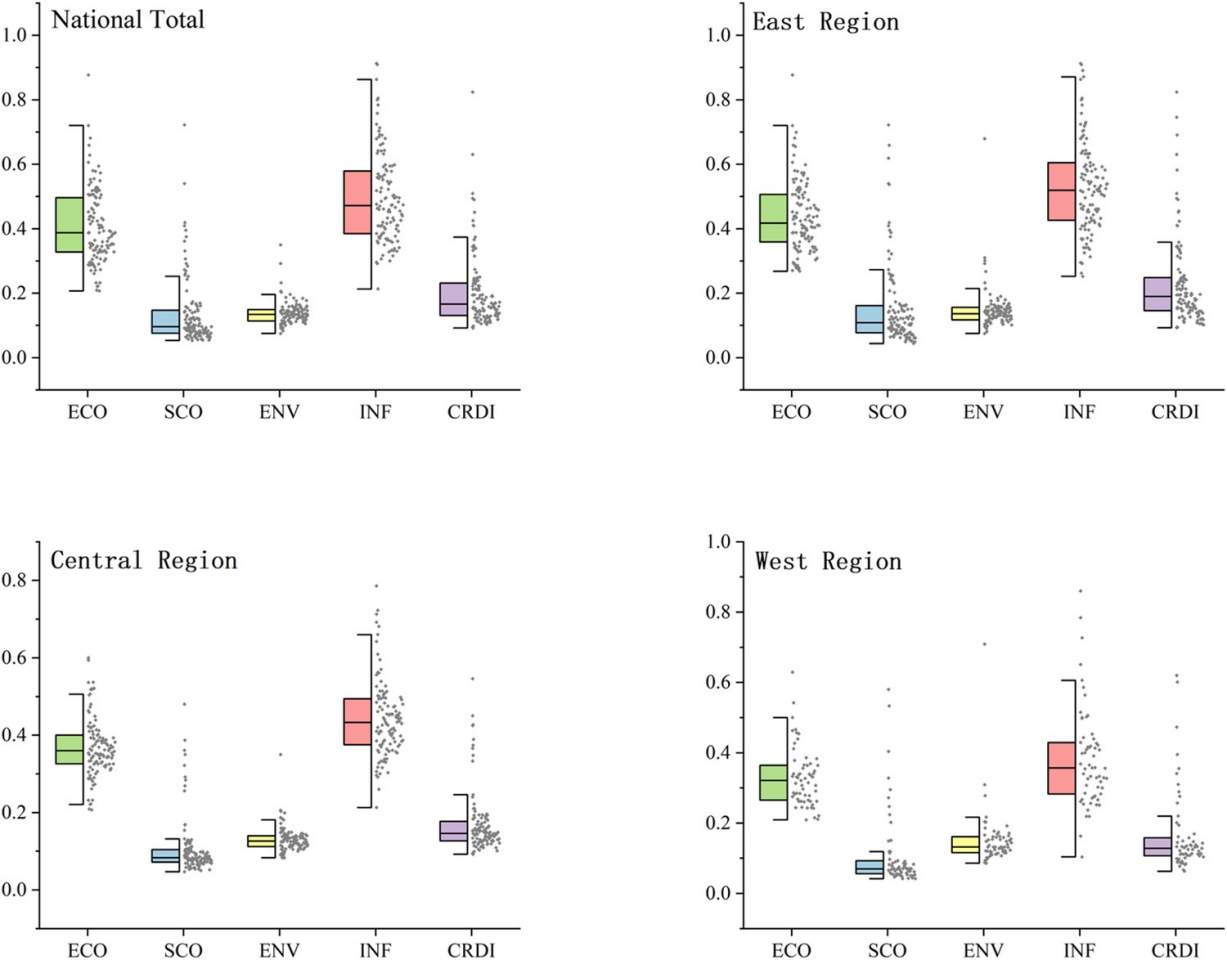
Distribution of the total CRDI index with sub-dimensions for the whole country and the three sub-regions.

The mean, standard deviation, coefficient of variation, and median performance of the national and three major sub-divisions are shown in S2 Table in S1 File. The regional sub-dimension distribution is shown in Fig 7. From the comprehensive icon results, the development of ECO and INF is in the order of east, central, and west from high to low. ENV is close to SCO in the central and western regions. The median ENV is higher in the central region than in the western region. The coefficient of variation is smaller than that in the western region, which shows that the overall level of the central region is better than that in the western region. In ENV, the median of the eastern region is higher than the western region. The coefficient of variation is smaller than in the western region, which shows that the general level of ENV in the eastern region is better than that in the western region, and the eastern region has more balanced development than the western region. Therefore, the levels of economic, social, and infrastructure resilience are from high to low in the eastern, central, and western regions, while the levels of ENV are from high to low in the eastern, western, and central regions. SCO is a shortcoming area in the three regions.

### 4.3 CRDI coupling coordination analysis

**Fig 8:**
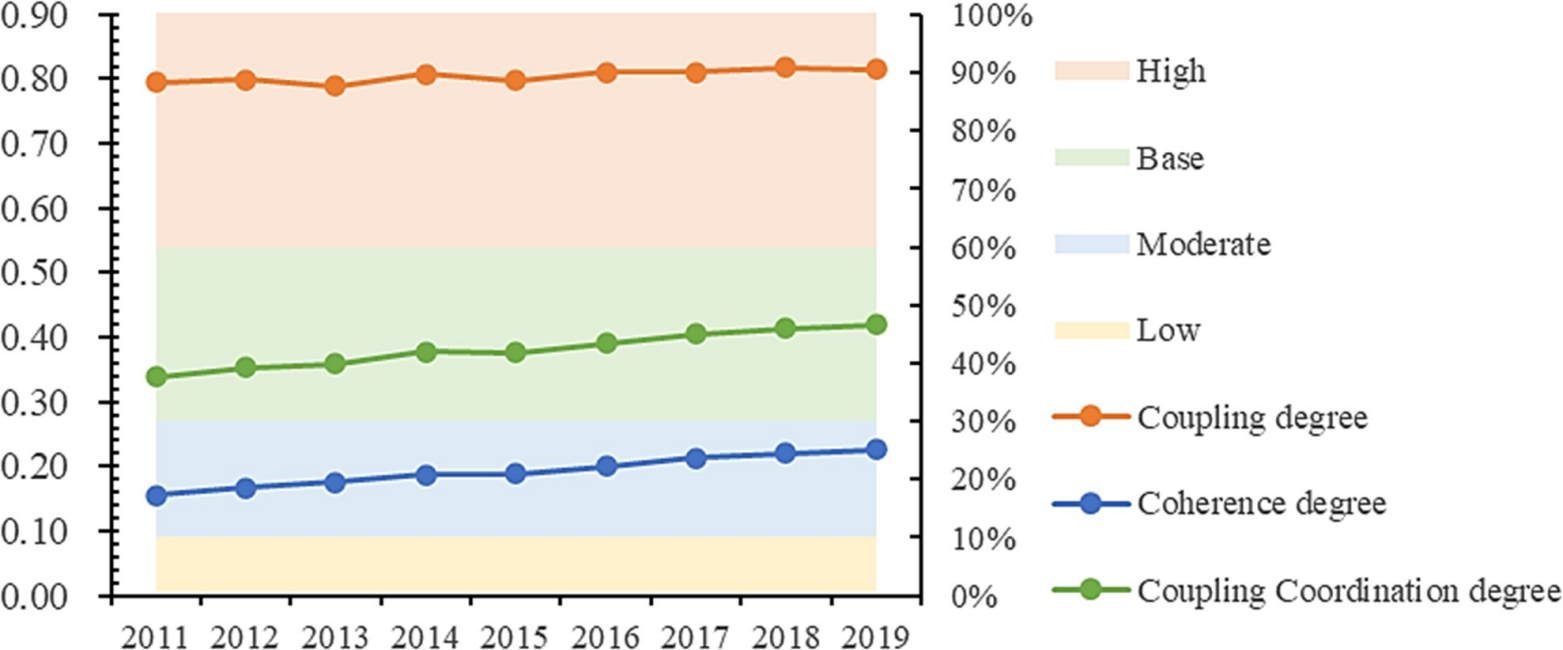
Time trend of CRDI coupling coordination.

Table 6 indicates the calculation results of the coupling coordination degree of CRDI subsystems. Combined with Fig 8, the results show that the coupling degree, coordination degree, and coupling coordination degree among CRDI sub-dimensions from 2011 to 2019 have indicated an upward trend. The CRDI coupling degree in China from 2011 to 2019 is around 0.8, which is a highly coupled state overall, which shows that the connection between CRDI subsystems in China is relatively close, and closeness is increasing. The coordination degree is between 0.1 and 0.3, and it changes from low coordination to medium coordination, and the coupling coordination degree is also in a rising trend. The results of the coupling degree, coordination degree, and coupling coordination degree indicators show a mutually constraining coupling relationship within CRDI indicators. In 2012, China put forward the overall layout concept of joint construction of economic, political, cultural, social, and ecological five directions, which to a certain extent, makes the coupling degree, coordination degree, and coupling coordination degree of CRDI subsystem indicate an increasing trend.

**Table 6:** Distribution of CRDI subsystem coupling coordination index levels.

Year	Coupling	Coordination	Coupling coordination	Level
	degree	index	degree	
2011	0.795	0.156	0.339	Mildly disordered
2012	0.800	0.168	0.353	Mildly disordered
2013	0.789	0.175	0.359	Mildly disordered
2014	0.807	0.188	0.377	Mildly disordered
2015	0.798	0.189	0.376	Mildly disordered
2016	0.810	0.200	0.391	Mildly disordered
2017	0.810	0.213	0.405	On the verge of disorder
2018	0.818	0.221	0.414	On the verge of disorder
2019	0.816	0.227	0.420	On the verge of disorder

However, regions with solid economic resilience usually attract more labor force, thus enhancing regional population density. In contrast, densely populated regions have high social demands, which to a certain extent, promotes regional SCO development and puts pressure on INF and ENV. It is worth mentioning that the urban resilience reference only appeared in 2021. Before that, the regional attention to resilience was low, so the regional coordination level in promoting a resilient system could be higher.

### 4.4 Barrier factor analysis

**Fig 9:**
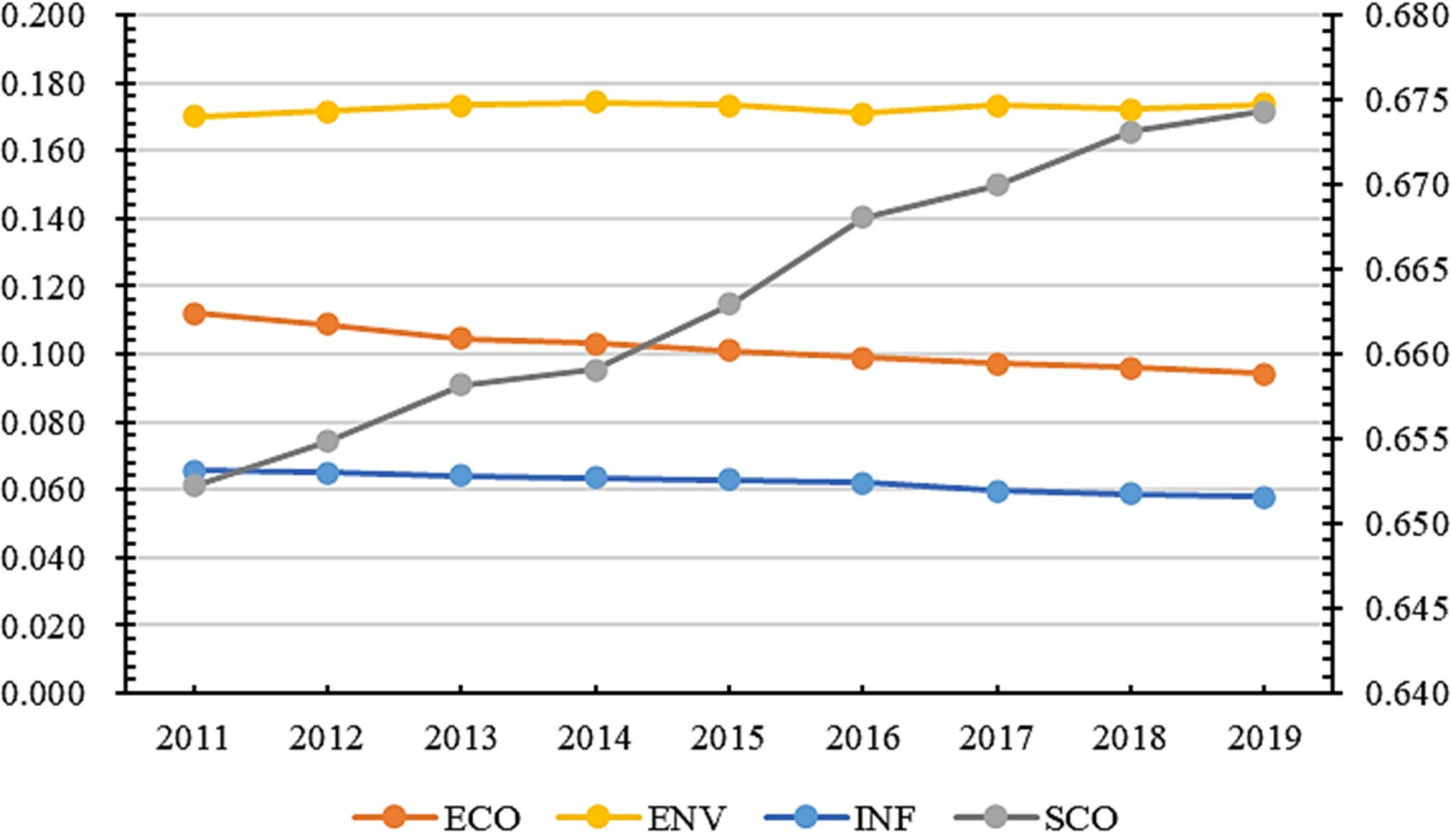
Temporal trend of CRDI guideline layer barrier degree.

The barrier degree of each criterion level to ASDI and the trend of change are shown in Fig 9. From the viewpoint of barrier strength, the barrier degree is ranked from high to low: INF < ECO < ENV < SCO, showing that social resilience is the primary constraint for improving CRDI. From the evolution trend of barrier degree, the barrier infrastructure resilience, economic resilience, and environmental resilience indicate a decreasing trend. In contrast, the barrier degree of social resilience indicates an increasing trend. Therefore, improving social resilience is a significant breakthrough in promoting the growth of CRDI.

The following explanations are given for the mechanisms of the four dimensions’ influence on urban resilience development. Social resilience refers to a region’s ability to maintain social integration, ensure social governance, and keep society functioning effectively when social structures are exposed to shocks or risks. First, the overall social resilience level needs to be stronger in China’s urban resilience. On the one hand, as the population rises and people’s income increases, the demand for social services to establish a social service system that meets the needs of society is much faster than the supply. On the other hand, to realize the scale efficiency of social public service resources, urban social development often shows a "high-high-low-low" state. The more populated and developed areas are usually densely populated with social resources, making social resources suffer significant trauma when facing risks. The workload to restore the original level of social services to meet the needs of residents is enormous. The workload is huge. Second, SCO hinders the overall development of urban resilience, and the supply of social services cannot keep up with demand, which to some extent, reduces the well-being of residents’ lives and leads to low motivation of residents to create wealth to resist risks. At the same time, public goods generate competition when resources are limited, which leads to an unbalanced allocation of social service resources, which can make residents less conscious of using public goods, leading to a significant difference in the quality of life of residents and low social cohesion, thus hindering the development of overall urban resilience. Currently, people’s demand and requirement level for medical care, education, security, and living elements are gradually increasing. The hindering effect of SCO has made a rising trend in recent years in the face of the characteristics of SCO enhancement with a long period and high cost.

In ECO, establishing a community of human destiny and an open economic development model have elevated the risks accompanying economic development. In the face of such a situation, China has optimized and upgraded its industrial structure, expanded innovation, and created new industries. For example, Shenzhen has vigorously promoted the development of new industries by grasping global technology trends and accelerated the establishment of a global science and technology innovation center; Hangzhou has seized the opportunity of rapid development of the Internet, encouraged innovation in Internet-based technologies and business models, and become a vibrant "Internet capital. At the same time, China’s strategy of expanding domestic demand, which is actively promoted, accelerates factor market-oriented reforms, promotes the efficient and smooth flow of factor resources, including labor, and optimizes the allocation according to economic development needs, to a certain extent, promotes the development of ECO, thus making the level of ECO hindrance decrease.

In ENV, with economic development, population agglomeration will put pressure on the ecosystem of cities. However, in recent years, China has put forward the concept of green and sustainable development, introduced the system of property rights of natural resources assets, the system of paid use of resources, the system of ecological compensation, the system of evaluation and assessment of ecological civilization performance and accountability, and other ecological protection systems, which have improved the efficiency of pollution control and raised the awareness of environmental protection including enterprises and residents and enhanced the ecological resilience of the region. Therefore, the hindering effect of ENV on urban resilience has been gradually reduced.

In the field of INF with the progress of the times, and the diversified development of people’s economic life, the demand for infrastructure and resources is also rising. China current China is actively promoting the construction of a modern comprehensive three-dimensional transportation system and modern energy system work, and the overall level of infrastructure has been significantly improved. By the end of 2021, the total mileage of China’s comprehensive transportation network exceeded 6 million kilometers, 843,000 kilometers of 220 kV, and above transmission lines. The total length of fiber optic cable lines reached 54.81 million kilometers, equivalent to 1.3 times 1.7 times, and 3.7 times ten years ago, respectively. The total reservoir capacity reached 903.5 billion cubic meters, high-speed rail, highways, power grids, 4G network size, and other long-term stability worldwide—the first. The perfection of China’s infrastructure construction is improving, which is also improving the INF, thus increasing the degree of CRDI.

**Fig 10:**
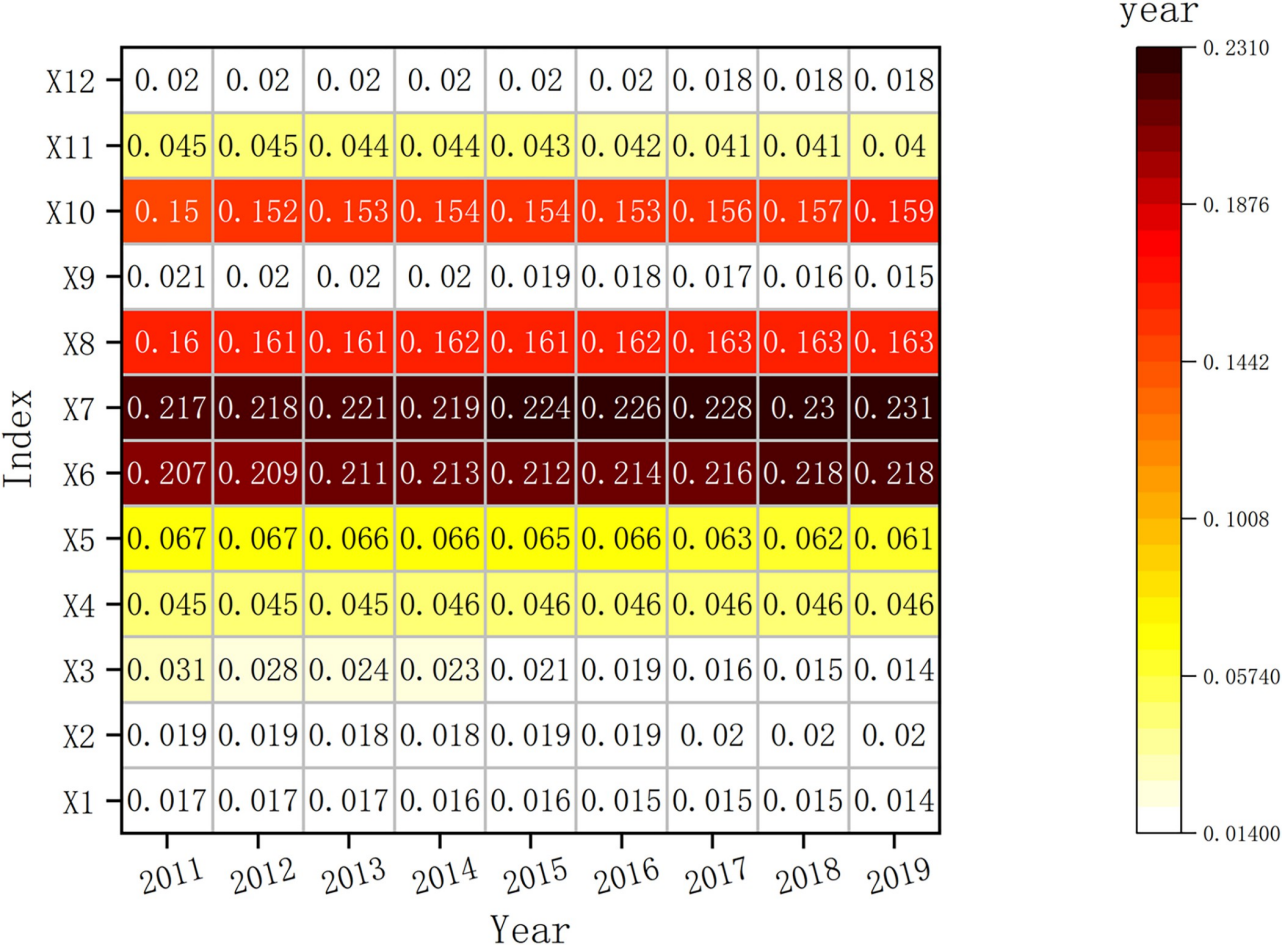
Temporal trend of CRDI indicator layer barrier degree.

Fig 10 and S3 Table in S1 File indicate the barrier degree of each indicator layer of CRDI. The heat map shows that the barrier degree is X7 > X6 > X8 > X10 > X5 > X4 > X11 > X3 > X12 > X2 > X9 > X1 in descending order. The results indicate that social security, social education, social life, environmental protection, and social and medical care are the main barrier factors. From a dynamic perspective, X1, X3, X5, X9, X11, and X12 indicate a decreasing trend from 2011 to 2019, while X2, X4, X6, X7, X8, and X10 indicate an increasing trend, which shows that the barriers to the workforce, innovation, education, security, social life, and environmental protection to CRDI are gradually increasing, so improving the optimization measures for these six areas is necessary.

## 5. Discussion

### 5.1 Regional and country level analysis

This paper analyzes the level of resilient urban development in China at the overall and regional levels spatially and temporally, taking cities and sample data from 2011 to 2019 as examples. First, overall, China’s CRDI level is low, and there is uneven development in four major resilience development areas: ECO, SCO, ENV, and INF, which is mainly due to the low importance and late start of resilient city construction in China during the sample period. Over time, CRDI has shown an increasing trend due to the increasing importance of CRDI construction in China, the strengthening of crisis and bottom-line awareness, and the real CRDI improvement showing an upward trend. Sub-dimension also shows an upward trend. ECO rises because of the continuous progress of productivity, the emergence of high-efficiency production technology, and the introduction of advanced foreign technology and new industries under the open pattern, which promotes regional economic development and enhances ECO. INF and SCO are rising because economic development has led to the continuous development of infrastructure and vital energy. There are also higher requirements for the quality of the labor force, the increase in population, people’s demand for a better quality of life, and high energy demand. ENV has increased because regional economic development and population concentration have put significant pressure on ecology, leading to a growing awareness of environmental protection and introducing environmental regulation policies to improve ecological quality in recent years, increasing ecological resilience.

Second, regionally, the total CRDI index is from high to low in the order of East, Central, and West. In the sub-dimensions, the ECO, SCO, and INF are high to low in the order of East, Central, and West. The ENV is high to low in the order of East, West, and Central due to the relatively open geographical location of the eastern region that makes its facilities developed. The high level of openness can introduce cash ideas and industries, attracting labor force employment and relatively rich living resources. Its resources are also more competitive compared to the eastern region. The development of the inland central and western regions by geographical constraints, and resources than coastal areas, especially the western region, the development more rely on a high policy tilt, and thus In, CRDI, ECO, SCO, and IVF show a decreasing spatial layout from coastal to inland. However, regarding ENV, the eastern region has achieved green and sustainable development faster by introducing eco-friendly industries and technologies. In contrast, regional development while the western region has to be due to the central region because the heavy industrial and population pressure in the western region is less than that in the central region, which makes the ecological pressure relatively weak. In the face of risks, the ecology of the western region is better than that of the central region in terms of resilience. Thus, the ENV of the East, West, and Central ranking situation.

Thirdly, there is a positive spatial spillover effect of CRDI and its sub-dimensions on regional spatial distribution, which is mainly analyzed from two aspects. On the one hand, the neighboring regions have exemplary effects on local development, which can lead to the CRDI development of the region and stimulate the learning effect of the region, thus improving the resilience level of the region. On the other hand, the CRDI development of neighboring regions can enhance the competitiveness of neighboring regions in terms of resources or even cause the loss of resources in the region, which will stimulate the crisis consciousness of the region and thus enhance its urban resilience construction, thus enhancing the level of resilience of the region. This applies to ECO, SCO, and INF. It is worth mentioning that the increased awareness of environmental protection in neighboring regions will promote the ENV of neighboring regions, which will also bring a crowding out effect to the heavily polluting enterprises in neighboring regions, and in order to prevent the pollution sanctuary effect, the local area will also improve its own environmental regulation level, thus improving ENV. Therefore, CRDI and its sub-dimensions show a positive spatial spillover effect.

Fourth, in the coupling coordination degree analysis, the coupling coordination degree of the CRDI system shows an increasing trend from 2011 to 2019. However, it is still at the edge of disorder, which indicates that China needs to pay more attention to urban resilience development during the sample period. The joint development of CRDI still needs improvement. Meanwhile, there are also contradictory points of development within CRDI, such as economic development sometimes cannot consider ecological protection, and promoting SCO construction will bring massive pressure on essential resources. Therefore, the coupling coordination course is still at the edge of disorder. However, with the deepening of China’s concept of regional sustainable development, the coupling coordination degree of the CRDI system is continuously improving.

Finally, SCO in the criteria tier is the main barrier factor in the barrier factor analysis. Human resources, innovation, education, security, and living in the indicator tier are the key areas that need to be improved in the collaborative development of CRDI. As the population increases, people’s quality of life and living resources are more demanding, requiring the region to provide living services and education to match. At the same time, economic growth brings population clustering at a rate higher than the rate of social provision of services. Social service development then hinders the overall resilience of the region. Population resources, education, innovation, security, life, and environmental protection are interrelated at the indicator level. A low level of social security will reduce the attractiveness of the labor force, and a low level of education will lead to a low quality of the labor force and, thus, a low innovation of the labor force, which are all indicators of SCO.

### 5.2 Case analysis

This section takes Nanjing as an example to further illustrate the applicability of this study. Nanjing was chosen because, unlike the individual policies and resources of provincial-level municipalities, it is a city with overall sustainable development and has few characteristics in terms of urban hierarchy. At the same time, in recent years, the concept of resilient city building in Nanjing has gradually increased. Meetings have been conducted to listen to various suggestions and deploy effective initiatives for risk prevention and resilience enhancement. Therefore, Nanjing, for example, has a certain typicality.

**Fig 11:**
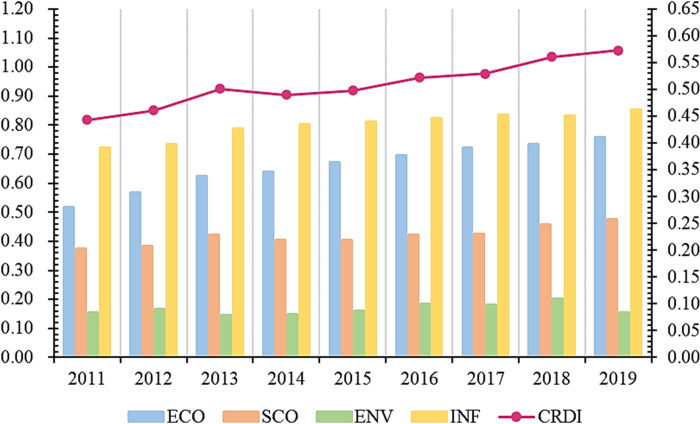
CRDI time trend of Nanjing.

The total CRDI index and sub-dimensional indicators of Nanjing are shown in Fig 11. The overall level of urban resilience, Nanjing’s CRDI, is on an upward trend. Combined with the actual development of Nanjing, on the first working day after the Spring Festival in 2018, Nanjing held its first meeting to determine the goals of coordinated development in eight major areas, including innovation, industry, ecology, reform, openness, and people’s livelihood. After that, on the first working day after the Spring Festival in 2019, 2020, and 2021, Nanjing will hold a meeting to accept the achievements of the current year, which will help Nanjing’s overall development to a certain extent. The overall indicator also shows that Nanjing’s urban resilience grew from 0.561 to 0.573 from 2018 to 2019.

From the perspective of ECO, on the one hand, Nanjing is in the coastal zone and close to Shanghai, with vital openness, regional advantages, and solid regional radiation, improving Nanjing’s economic resilience level. Nanjing is an essential comprehensive industrial production base in China, with electronic information, petrochemicals, iron, and steel dominated mainly by state-owned enterprises and large enterprises. To a certain extent, it brings a dynamic industrial development environment for Nanjing, attracting investment and promoting innovation. From the estimation results, Nanjing’s economic resilience increased from 0.517 to 0.760 from 2011 to 2019.

From the perspective of ENV, in 2013, Nanjing’s Environmental Protection Administration received strong reactions from the public, and there were inconsistencies between the city’s air testing results and the public’s perceptions. To achieve the Blue Sky Plan in 2013, Nanjing changed the air testing standards (AQI). Compared with the old standards (API), the new standards will be delicate particulate matter (PM2.5), ozone (O3), and carbon monoxide (CO) the measurement of three, and the measurement results will be released once a day instead of once every hour, which has stimulated environmental awareness in Nanjing to some extent. From the environmental toughness index, the results show that before 2013, Nanjing’s environmental toughness index indicated a decreasing trend, which to some extent, shows that the use of API as a testing standard makes Nanjing’s assessment of the actual quality of the environment inaccurate. After the enhanced air testing in 2013, Nanjing’s environmental toughness index indicated an upward trend.

From the perspective of social and infrastructural resilience, in 2010, Nanjing’s local government issued the Proposal of the CPC Nanjing Municipal Committee on the 12th Five-Year Plan. It proposed to build a happy city for the people as the fundamental goal, promote social construction focusing on improving people’s livelihood, and improve social security, social education, social and medical care, public safety, family services, and other social and infrastructural systems. The 12th Five-Year Plan is proposed to promote social construction, focusing on improving people’s livelihood and social security, social education, social, and medical care, public safety, transportation, and family services, which has led the Nanjing government to emphasize social and infrastructure construction. Nanjing’s urban resilience is on an upward trend from 2011 to 2019, with social resilience rising from 0.376 to 0.475 and essential resilience rising from 0.443 to 0.573.

### 5.3 Research contributions and relevance to real-world problems

The findings of this paper have profound theoretical and practical implications. In terms of theoretical significance, compared with the current research results, this paper makes the following contributions to the field of urban resilience. First, it contributes Chinese empirical evidence in urban resilience research. Unlike the existing analysis of resilience development in cross-sectional data in China and the analysis of resilience development in a particular province and city in China, this paper uses panel data from 284 cities across China to conduct a spatial and temporal analysis of urban resilience from 2011 to 2019. Second, the theoretical foundation of this paper is based on the framework of sustainable development, which makes this paper use the systemic analysis method to analyze four aspects of urban human nature: economic, social, raw energy, and ecological, and the research results of this paper are more comprehensive than the research results of resilience analysis in one field. Third, this paper introduces the coupling coordination degree and barrier factor analysis to analyze further the collaborative development of various fields in China under the urban resilience framework, further highlighting the systematic analysis thinking of this paper.

Practical significance. First, the reasons for the findings are explained in the context of China’s current development characteristics. At the same time, the case study of Nanjing is used to confirm the reasonableness of the findings at the display level. Secondly, it is possible to identify the regions and areas that need to be improved in the coordinated development process based on the current overall and regional spatial and temporal conditions of urban resilience in China. Finally, the theoretical framework applied in this paper is aligned with the United Nations. It revolves around the current hot issues of sustainable development in the United Nations, and its research framework and systematic analysis are also of reference value in the international context.

## 5 Conclusions and policy recommendations

This paper uses the entropy method to calculate the CRDI of 284 cities in China from 2011 to 2019. It analyzes the indexes by combining the coupling coordination degree and barrier factor analysis to obtain the following conclusions: (1) At the national level, the overall development level of CRDI is low. CRDI and its sub-dimensions indicate a growth trend in time and a decreasing spatial layout from coastal to inland in space. CRDI and sub-dimensions indicate a decreasing spatial layout from coastal to inland and a “high-high-low-low” clustering characteristic. (2) At the regional level, the CRDI is from high to low in the east, middle, and west order. The sub-dimensions are from high to low in the order of east, middle, and west for ECO, SCO, and INF and from high to low in the order of east, west, and middle for ENV. (3) In the coupling coordination degree analysis, the coupling coordination degree of the CRDI system is increasing from 2011 to 2019, but it is still on the verge of disorder. (4) In the analysis of obstacle factors, SCO in the criterion layer is the main obstacle factor, and human resources, innovation, education, security, and living in the indicator layer are the key areas that need to be improved in the collaborative development of CRDI. Based on the above findings, the following suggestions are proposed to improve the development of the urban resilience level.

1. In the face of low urban resilience development, the government should focus on coordinating all components while strengthening bottom-line thinking. Accelerate industrial transformation, improve the institutional environment for regional private and entrepreneurship, provide more new economic growth points for the region, and protect and improve the self-healing ability of SMEs through financial subsidies, credit preferences, and government services. Actively leverage the construction of new infrastructure such as the Internet, 5G, and artificial intelligence to promote the intelligent transformation of industries. It improves the institutional environment for social demands, including social healthcare, education, infrastructure, security, and services, and effectively solves residents’ living problems. Some regions should consider the local environment’s carrying capacity in economic development. Increase the penetration of the green development concept, expand pilot environmental regulation policies, and actively use environmental regulation tools such as green loans, emission fees, and emission trading markets to strengthen regional environmental bottom-line thinking.
2. Facing the uneven regional development of CRDI, the government should actively coordinate regional differences. The eastern region should take advantage of its location to actively introduce advanced concepts and resources and be a role model to lead the central and western regions to imitate and learn. The central and western regions should actively explore their unique intrinsic value and economic growth points in their regional endowment, foundation, and other conditions. The government should actively guide the central and western regions to establish the correct development values and promote the balanced development of the regional urban resilience system.
3. In the face of uncoordinated development despite the close connection within urban resilience, the government should reasonably develop and layout urban construction and development resources, build bottom-line thinking, and improve inherent coordination among economic, social, ecological, and infrastructural construction. The government should establish a global concept, coordinate the development of economic, social, ecological, and infrastructure aspects, improve the regional social construction and infrastructure carrying capacity according to the actual regional development, implement the concept of sustainable development, and focus on the coordinated development of economy and ecology, to form a virtuous circle and realize the overall improvement of coupling and coordination.
4. Paying attention to social resilience is the main factor that hinders the overall development of the urban resilience system, especially the construction of three aspects: social security, social education, and social life. The government should improve the social insurance system, introduce advanced education concepts, and increase investment in public social goods in the face of rising population numbers. At the same time, facing the situation of obstacles to the workforce, innovation, and environmental protection to CRDI are increasing year by year. The government should optimize the policy of introducing talents, raising the awareness of property rights and improving the policy of subsidizing innovation to provide a suitable growth environment for innovation, strengthen environmental protection awareness and actively encourage enterprises to take their corresponding social responsibility.

## Supporting information

S1 File (DOCX)
