# Supplementary material for: Spatial and temporal evolution of urban resilience in China and analysis of barriers: Based on a sustainable development perspective
Source: PLoS One. 2024 Feb 6;19(2):e0285113. doi: 10.1371/journal.pone.0285113 (PMC10846694; doi:10.1371/journal.pone.0285113)
Supplement: S1 File — (DOCX) [file pone.0285113.s001.docx]

Appendix

S1 Table. Distribution table of CRDI and its sub-dimensions (in descending order of CRDI size)

| number | city | ECO | SCO | ENV | INF | CRDI |
| --- | --- | --- | --- | --- | --- | --- |
| 1 | Beijing | 0.877 | 0.722 | 0.292 | 0.913 | 0.824 |
| 2 | Shenzhen | 0.699 | 0.659 | 0.310 | 0.871 | 0.746 |
| 3 | Guangzhou | 0.655 | 0.619 | 0.214 | 0.891 | 0.691 |
| 4 | Shanghai | 0.720 | 0.540 | 0.176 | 0.909 | 0.630 |
| 5 | Chongqing | 0.463 | 0.580 | 0.166 | 0.860 | 0.620 |
| 6 | Chengdu | 0.629 | 0.533 | 0.186 | 0.784 | 0.601 |
| 7 | Dongguan | 0.540 | 0.537 | 0.159 | 0.730 | 0.582 |
| 8 | Wuhan | 0.600 | 0.480 | 0.146 | 0.786 | 0.546 |
| 9 | Nanjing | 0.659 | 0.419 | 0.166 | 0.800 | 0.509 |
| 10 | Tianjin | 0.606 | 0.409 | 0.142 | 0.863 | 0.494 |
| 11 | Hangzhou | 0.681 | 0.395 | 0.162 | 0.784 | 0.490 |
| 12 | Xi’an | 0.542 | 0.404 | 0.162 | 0.727 | 0.473 |
| 13 | Jinan | 0.566 | 0.388 | 0.132 | 0.705 | 0.455 |
| 14 | Xiamen | 0.573 | 0.375 | 0.177 | 0.680 | 0.451 |
| 15 | Zhengzhou | 0.537 | 0.387 | 0.119 | 0.723 | 0.450 |
| 16 | Changsha | 0.521 | 0.350 | 0.199 | 0.681 | 0.427 |
| 17 | Taiyuan | 0.536 | 0.361 | 0.148 | 0.642 | 0.425 |
| 18 | Zhuhai | 0.598 | 0.322 | 0.267 | 0.633 | 0.423 |
| 19 | Shenyang | 0.553 | 0.331 | 0.142 | 0.724 | 0.410 |
| 20 | Suzhou | 0.628 | 0.306 | 0.151 | 0.805 | 0.409 |
| 21 | Kunming | 0.500 | 0.328 | 0.160 | 0.651 | 0.395 |
| 22 | Nanchang | 0.519 | 0.322 | 0.157 | 0.609 | 0.389 |
| 23 | Hefei | 0.594 | 0.284 | 0.134 | 0.713 | 0.374 |
| 24 | Haerbin | 0.518 | 0.292 | 0.138 | 0.660 | 0.366 |
| 25 | Qingdao | 0.573 | 0.256 | 0.190 | 0.718 | 0.358 |
| 26 | Wulumuqi | 0.454 | 0.295 | 0.144 | 0.606 | 0.355 |
| 27 | Changchun | 0.506 | 0.269 | 0.140 | 0.692 | 0.348 |
| 28 | Sanya | 0.410 | 0.202 | 0.679 | 0.381 | 0.347 |
| 29 | Dalian | 0.544 | 0.261 | 0.140 | 0.685 | 0.346 |
| 30 | Shijiazhuang | 0.506 | 0.273 | 0.104 | 0.679 | 0.344 |
| 31 | Guiyang | 0.466 | 0.272 | 0.177 | 0.586 | 0.341 |
| 32 | Ningbo | 0.578 | 0.243 | 0.156 | 0.703 | 0.340 |
| 33 | Fuzhou | 0.549 | 0.252 | 0.156 | 0.643 | 0.339 |
| 34 | Foshan | 0.532 | 0.241 | 0.168 | 0.724 | 0.336 |
| 35 | Huhehaote | 0.504 | 0.256 | 0.187 | 0.556 | 0.333 |
| 36 | Zhongshan | 0.555 | 0.255 | 0.157 | 0.524 | 0.331 |
| 37 | Haikou | 0.458 | 0.230 | 0.301 | 0.539 | 0.324 |
| 38 | Wuxi | 0.580 | 0.207 | 0.149 | 0.758 | 0.315 |
| 39 | Lanzhou | 0.457 | 0.247 | 0.132 | 0.564 | 0.310 |
| 40 | Nanning | 0.462 | 0.234 | 0.165 | 0.605 | 0.309 |
| 41 | Yinchuan | 0.439 | 0.224 | 0.171 | 0.506 | 0.289 |
| 42 | Kelamayi | 0.364 | 0.202 | 0.309 | 0.408 | 0.276 |
| 43 | Changzhou | 0.555 | 0.173 | 0.134 | 0.690 | 0.274 |
| 44 | Wenzhou | 0.528 | 0.167 | 0.156 | 0.632 | 0.264 |
| 45 | Jiayuguan | 0.279 | 0.119 | 0.709 | 0.300 | 0.257 |
| 46 | Yantai | 0.513 | 0.161 | 0.156 | 0.621 | 0.255 |
| 47 | Quanzhou | 0.505 | 0.161 | 0.146 | 0.598 | 0.250 |
| 48 | Shaoxing | 0.528 | 0.159 | 0.125 | 0.594 | 0.248 |
| 49 | Daqing | 0.459 | 0.168 | 0.145 | 0.561 | 0.246 |
| 50 | Weihai | 0.464 | 0.150 | 0.213 | 0.559 | 0.244 |
| 51 | Weifang | 0.489 | 0.164 | 0.117 | 0.585 | 0.244 |
| 52 | Jiaxing | 0.514 | 0.164 | 0.125 | 0.522 | 0.244 |
| 53 | Huizhou | 0.464 | 0.151 | 0.179 | 0.582 | 0.241 |
| 54 | Baotou | 0.463 | 0.154 | 0.172 | 0.562 | 0.240 |
| 55 | Jinhua | 0.522 | 0.150 | 0.142 | 0.545 | 0.239 |
| 56 | Zhenjiang | 0.496 | 0.142 | 0.163 | 0.584 | 0.236 |
| 57 | Zibo | 0.462 | 0.144 | 0.144 | 0.632 | 0.233 |
| 58 | Xuzhou | 0.506 | 0.139 | 0.121 | 0.641 | 0.232 |
| 59 | Baoding | 0.438 | 0.158 | 0.115 | 0.570 | 0.231 |
| 60 | Nantong | 0.520 | 0.125 | 0.148 | 0.664 | 0.229 |
| 61 | Zhoushan | 0.402 | 0.145 | 0.232 | 0.433 | 0.223 |
| 62 | Liuan | 0.383 | 0.169 | 0.136 | 0.426 | 0.222 |
| 63 | Tangshan | 0.452 | 0.141 | 0.101 | 0.634 | 0.222 |
| 64 | Xining | 0.383 | 0.151 | 0.164 | 0.502 | 0.220 |
| 65 | Taizhou | 0.496 | 0.121 | 0.149 | 0.578 | 0.215 |
| 66 | Jiangmen | 0.473 | 0.129 | 0.128 | 0.557 | 0.213 |
| 67 | Yangzhou | 0.499 | 0.117 | 0.143 | 0.598 | 0.213 |
| 68 | Qinhuangdao | 0.387 | 0.144 | 0.147 | 0.518 | 0.212 |
| 69 | Huzhou | 0.475 | 0.122 | 0.155 | 0.539 | 0.210 |
| 70 | Luoyang | 0.473 | 0.127 | 0.119 | 0.570 | 0.210 |
| 71 | Wuhu | 0.519 | 0.117 | 0.110 | 0.595 | 0.210 |
| 72 | Yichang | 0.449 | 0.125 | 0.140 | 0.504 | 0.203 |
| 73 | Panzhihua | 0.333 | 0.149 | 0.148 | 0.429 | 0.201 |
| 74 | Linyi | 0.406 | 0.121 | 0.112 | 0.632 | 0.200 |
| 75 | Liuzhou | 0.439 | 0.119 | 0.140 | 0.538 | 0.200 |
| 76 | Jining | 0.441 | 0.124 | 0.098 | 0.560 | 0.199 |
| 77 | Zhuzhou | 0.480 | 0.111 | 0.132 | 0.527 | 0.196 |
| 78 | Guilin | 0.411 | 0.122 | 0.168 | 0.461 | 0.196 |
| 79 | Benxi | 0.306 | 0.147 | 0.158 | 0.416 | 0.195 |
| 80 | Taian | 0.425 | 0.124 | 0.111 | 0.521 | 0.195 |
| 81 | Langfang | 0.439 | 0.127 | 0.109 | 0.470 | 0.195 |
| 82 | Nanyang | 0.388 | 0.122 | 0.139 | 0.539 | 0.194 |
| 83 | Mianyang | 0.427 | 0.119 | 0.132 | 0.515 | 0.194 |
| 84 | Ganzhou | 0.421 | 0.123 | 0.124 | 0.501 | 0.194 |
| 85 | Handan | 0.429 | 0.118 | 0.105 | 0.559 | 0.194 |
| 86 | Xiangtan | 0.393 | 0.130 | 0.109 | 0.498 | 0.193 |
| 87 | Dongying | 0.438 | 0.108 | 0.158 | 0.532 | 0.193 |
| 88 | Erdos | 0.423 | 0.104 | 0.206 | 0.494 | 0.193 |
| 89 | Yancheng | 0.479 | 0.100 | 0.137 | 0.567 | 0.192 |
| 90 | Shiyan | 0.366 | 0.120 | 0.181 | 0.457 | 0.190 |
| 91 | Taizhou | 0.473 | 0.100 | 0.128 | 0.579 | 0.190 |
| 92 | Huaian | 0.449 | 0.101 | 0.132 | 0.592 | 0.190 |
| 93 | Jilin | 0.393 | 0.120 | 0.106 | 0.526 | 0.187 |
| 94 | Xinxiang | 0.387 | 0.129 | 0.090 | 0.473 | 0.186 |
| 95 | Wuhai | 0.288 | 0.100 | 0.350 | 0.384 | 0.185 |
| 96 | Xiangfan | 0.439 | 0.106 | 0.135 | 0.508 | 0.185 |
| 97 | Jinzhong | 0.364 | 0.132 | 0.109 | 0.420 | 0.183 |
| 98 | Shantou | 0.396 | 0.103 | 0.129 | 0.592 | 0.183 |
| 99 | Fushun | 0.335 | 0.119 | 0.145 | 0.496 | 0.182 |
| 100 | Hengyang | 0.400 | 0.112 | 0.109 | 0.513 | 0.180 |
| 101 | Zhaoqing | 0.404 | 0.106 | 0.134 | 0.502 | 0.180 |
| 102 | Panjin | 0.330 | 0.109 | 0.196 | 0.452 | 0.178 |
| 103 | Jiujiang | 0.419 | 0.104 | 0.131 | 0.475 | 0.177 |
| 104 | Zhanjiang | 0.395 | 0.101 | 0.150 | 0.503 | 0.177 |
| 105 | Huangshi | 0.424 | 0.100 | 0.116 | 0.514 | 0.175 |
| 106 | Cangzhou | 0.415 | 0.108 | 0.097 | 0.460 | 0.173 |
| 107 | Jiaozuo | 0.402 | 0.107 | 0.105 | 0.475 | 0.172 |
| 108 | Zhangzhou | 0.428 | 0.096 | 0.131 | 0.474 | 0.172 |
| 109 | Anshan | 0.388 | 0.101 | 0.116 | 0.514 | 0.171 |
| 110 | Lianyungang | 0.434 | 0.086 | 0.122 | 0.552 | 0.169 |
| 111 | Xianyang | 0.369 | 0.115 | 0.107 | 0.413 | 0.169 |
| 112 | Anyang | 0.392 | 0.104 | 0.088 | 0.514 | 0.169 |
| 113 | Tongling | 0.371 | 0.106 | 0.149 | 0.410 | 0.169 |
| 114 | Maanshan | 0.432 | 0.093 | 0.114 | 0.498 | 0.169 |
| 115 | Dezhou | 0.420 | 0.093 | 0.103 | 0.526 | 0.167 |
| 116 | Jinzhou | 0.345 | 0.121 | 0.095 | 0.399 | 0.167 |
| 117 | Lishui | 0.407 | 0.086 | 0.184 | 0.426 | 0.165 |
| 118 | Changde | 0.412 | 0.087 | 0.143 | 0.480 | 0.165 |
| 119 | Binzhou | 0.404 | 0.091 | 0.097 | 0.533 | 0.164 |
| 120 | Shangqiu | 0.377 | 0.104 | 0.094 | 0.462 | 0.163 |
| 121 | Kaifeng | 0.410 | 0.099 | 0.090 | 0.454 | 0.162 |
| 122 | Heze | 0.369 | 0.102 | 0.081 | 0.507 | 0.162 |
| 123 | Yueyang | 0.402 | 0.087 | 0.130 | 0.492 | 0.162 |
| 124 | Deyang | 0.378 | 0.097 | 0.120 | 0.443 | 0.161 |
| 125 | Shaoguan | 0.436 | 0.083 | 0.138 | 0.443 | 0.160 |
| 126 | Suqian | 0.424 | 0.075 | 0.126 | 0.547 | 0.159 |
| 127 | Jinzhou | 0.414 | 0.097 | 0.083 | 0.443 | 0.159 |
| 128 | Putian | 0.404 | 0.075 | 0.160 | 0.504 | 0.159 |
| 129 | Xinyu | 0.345 | 0.098 | 0.138 | 0.428 | 0.158 |
| 130 | Qvzhou | 0.412 | 0.083 | 0.129 | 0.474 | 0.158 |
| 131 | Qiqihaer | 0.326 | 0.099 | 0.126 | 0.473 | 0.158 |
| 132 | Zunyi | 0.365 | 0.093 | 0.132 | 0.453 | 0.158 |
| 133 | Bangbu | 0.410 | 0.082 | 0.117 | 0.495 | 0.157 |
| 134 | Zhangjiakou | 0.355 | 0.090 | 0.135 | 0.463 | 0.156 |
| 135 | Liaocheng | 0.386 | 0.088 | 0.090 | 0.516 | 0.156 |
| 136 | Zaozhuang | 0.385 | 0.086 | 0.099 | 0.514 | 0.155 |
| 137 | Huainan | 0.361 | 0.089 | 0.125 | 0.466 | 0.154 |
| 138 | Zhumadian | 0.365 | 0.092 | 0.114 | 0.450 | 0.154 |
| 139 | Mudanjiang | 0.348 | 0.096 | 0.134 | 0.399 | 0.154 |
| 140 | Pingdingshan | 0.380 | 0.096 | 0.100 | 0.410 | 0.153 |
| 141 | Dandong | 0.328 | 0.096 | 0.130 | 0.433 | 0.153 |
| 142 | Datong | 0.354 | 0.089 | 0.115 | 0.485 | 0.153 |
| 143 | Chengde | 0.341 | 0.090 | 0.157 | 0.409 | 0.153 |
| 144 | XIngtai | 0.388 | 0.092 | 0.075 | 0.476 | 0.153 |
| 145 | Maoming | 0.370 | 0.087 | 0.135 | 0.425 | 0.152 |
| 146 | Shangrao | 0.385 | 0.081 | 0.129 | 0.454 | 0.151 |
| 147 | Xinyang | 0.374 | 0.086 | 0.134 | 0.418 | 0.151 |
| 148 | Zhoukou | 0.360 | 0.090 | 0.106 | 0.452 | 0.150 |
| 149 | Yichun | 0.394 | 0.079 | 0.121 | 0.452 | 0.149 |
| 150 | Nanchong | 0.329 | 0.086 | 0.119 | 0.500 | 0.149 |
| 151 | Chenzhou | 0.382 | 0.079 | 0.140 | 0.430 | 0.149 |
| 152 | Fuyang | 0.376 | 0.083 | 0.093 | 0.496 | 0.149 |
| 153 | Anqing | 0.393 | 0.074 | 0.114 | 0.502 | 0.148 |
| 154 | Baoji | 0.339 | 0.086 | 0.132 | 0.439 | 0.147 |
| 155 | Rizhao | 0.378 | 0.075 | 0.107 | 0.517 | 0.147 |
| 156 | Chifeng | 0.346 | 0.082 | 0.128 | 0.461 | 0.146 |
| 157 | Changzhi | 0.350 | 0.091 | 0.105 | 0.410 | 0.146 |
| 158 | Longyan | 0.401 | 0.077 | 0.136 | 0.397 | 0.146 |
| 159 | Jingdezhen | 0.331 | 0.086 | 0.133 | 0.433 | 0.146 |
| 160 | Liaoyang | 0.295 | 0.091 | 0.123 | 0.459 | 0.146 |
| 161 | Ezhou | 0.345 | 0.082 | 0.145 | 0.412 | 0.145 |
| 162 | Yingkou | 0.359 | 0.082 | 0.112 | 0.453 | 0.145 |
| 163 | XIanning | 0.369 | 0.079 | 0.158 | 0.375 | 0.145 |
| 164 | Luzhou | 0.340 | 0.082 | 0.102 | 0.498 | 0.145 |
| 165 | Shaoyang | 0.358 | 0.082 | 0.120 | 0.436 | 0.144 |
| 166 | Beihai | 0.343 | 0.079 | 0.154 | 0.414 | 0.144 |
| 167 | Jieyang | 0.344 | 0.075 | 0.152 | 0.458 | 0.144 |
| 168 | Xiaogan | 0.373 | 0.082 | 0.105 | 0.434 | 0.144 |
| 169 | Hulunbeier | 0.328 | 0.094 | 0.143 | 0.317 | 0.144 |
| 170 | Shizuishan | 0.291 | 0.083 | 0.217 | 0.327 | 0.143 |
| 171 | Yuncheng | 0.334 | 0.096 | 0.091 | 0.374 | 0.142 |
| 172 | Xuchang | 0.380 | 0.080 | 0.106 | 0.415 | 0.142 |
| 173 | Linfen | 0.341 | 0.087 | 0.124 | 0.377 | 0.142 |
| 174 | Huanggang | 0.354 | 0.085 | 0.122 | 0.371 | 0.141 |
| 175 | Qingyuan | 0.369 | 0.073 | 0.117 | 0.461 | 0.140 |
| 176 | Chuzhou | 0.391 | 0.067 | 0.113 | 0.483 | 0.140 |
| 177 | Nanping | 0.378 | 0.077 | 0.155 | 0.325 | 0.140 |
| 178 | Meizhou | 0.376 | 0.069 | 0.145 | 0.419 | 0.139 |
| 179 | Ji’an | 0.369 | 0.071 | 0.140 | 0.426 | 0.139 |
| 180 | Jingmen | 0.344 | 0.072 | 0.135 | 0.447 | 0.138 |
| 181 | Sanming | 0.388 | 0.076 | 0.135 | 0.341 | 0.138 |
| 182 | Yanan | 0.331 | 0.091 | 0.131 | 0.302 | 0.138 |
| 183 | Qvjing | 0.386 | 0.075 | 0.113 | 0.391 | 0.138 |
| 184 | Yulin | 0.335 | 0.072 | 0.146 | 0.432 | 0.138 |
| 185 | Fuzhou | 0.352 | 0.069 | 0.137 | 0.454 | 0.138 |
| 186 | Huangshan | 0.349 | 0.065 | 0.203 | 0.361 | 0.137 |
| 187 | Yuxi | 0.364 | 0.069 | 0.169 | 0.358 | 0.136 |
| 188 | Hanzhong | 0.341 | 0.084 | 0.125 | 0.333 | 0.135 |
| 189 | Yaan | 0.282 | 0.090 | 0.154 | 0.307 | 0.135 |
| 190 | Xuancheng | 0.386 | 0.065 | 0.118 | 0.440 | 0.135 |
| 191 | Yangquan | 0.305 | 0.084 | 0.124 | 0.385 | 0.134 |
| 192 | Yulin | 0.348 | 0.072 | 0.119 | 0.422 | 0.133 |
| 193 | Tongliao | 0.325 | 0.076 | 0.130 | 0.404 | 0.133 |
| 194 | Chaozhou | 0.373 | 0.062 | 0.129 | 0.453 | 0.133 |
| 195 | Pingxiang | 0.349 | 0.075 | 0.124 | 0.383 | 0.133 |
| 196 | Leshan | 0.331 | 0.078 | 0.105 | 0.416 | 0.133 |
| 197 | Zigong | 0.411 | 0.066 | 0.121 | 0.368 | 0.133 |
| 198 | Huaihua | 0.348 | 0.076 | 0.117 | 0.384 | 0.132 |
| 199 | Weinan | 0.334 | 0.079 | 0.086 | 0.427 | 0.132 |
| 200 | Huaibei | 0.340 | 0.078 | 0.090 | 0.410 | 0.131 |
| 201 | Yongzhou | 0.341 | 0.073 | 0.114 | 0.398 | 0.130 |
| 202 | Yiyang | 0.314 | 0.071 | 0.124 | 0.444 | 0.130 |
| 203 | Tonghua | 0.332 | 0.077 | 0.136 | 0.328 | 0.130 |
| 204 | Yibin | 0.340 | 0.071 | 0.113 | 0.419 | 0.130 |
| 205 | Jiamusi | 0.290 | 0.078 | 0.134 | 0.377 | 0.129 |
| 206 | Fuxin | 0.270 | 0.087 | 0.112 | 0.369 | 0.128 |
| 207 | Luohe | 0.323 | 0.073 | 0.115 | 0.405 | 0.128 |
| 208 | Jiuquan | 0.307 | 0.070 | 0.203 | 0.277 | 0.128 |
| 209 | Suzhou | 0.353 | 0.068 | 0.083 | 0.455 | 0.127 |
| 210 | Puyang | 0.362 | 0.070 | 0.096 | 0.403 | 0.127 |
| 211 | Yangjiang | 0.331 | 0.062 | 0.126 | 0.459 | 0.127 |
| 212 | Sanmenxia | 0.342 | 0.074 | 0.109 | 0.354 | 0.126 |
| 213 | Ningde | 0.380 | 0.062 | 0.138 | 0.341 | 0.126 |
| 214 | Tongchuan | 0.315 | 0.071 | 0.153 | 0.329 | 0.125 |
| 215 | Liupanshui | 0.356 | 0.069 | 0.124 | 0.333 | 0.125 |
| 216 | Hegang | 0.209 | 0.083 | 0.185 | 0.316 | 0.125 |
| 217 | Baicheng | 0.271 | 0.076 | 0.158 | 0.333 | 0.125 |
| 218 | Baishan | 0.281 | 0.077 | 0.160 | 0.300 | 0.124 |
| 219 | Hengshui | 0.347 | 0.064 | 0.112 | 0.408 | 0.124 |
| 220 | Xinzhou | 0.288 | 0.082 | 0.092 | 0.370 | 0.124 |
| 221 | Siping | 0.300 | 0.080 | 0.093 | 0.364 | 0.123 |
| 222 | Jincheng | 0.333 | 0.069 | 0.113 | 0.361 | 0.122 |
| 223 | Guangyuan | 0.313 | 0.059 | 0.152 | 0.400 | 0.122 |
| 224 | Xinzhou | 0.281 | 0.071 | 0.119 | 0.409 | 0.121 |
| 225 | Loudi | 0.326 | 0.069 | 0.099 | 0.387 | 0.121 |
| 226 | Huludao | 0.286 | 0.071 | 0.106 | 0.420 | 0.121 |
| 227 | Jinchang | 0.209 | 0.068 | 0.278 | 0.219 | 0.120 |
| 228 | Wuzhou | 0.328 | 0.062 | 0.129 | 0.371 | 0.119 |
| 229 | Chizhou | 0.328 | 0.060 | 0.125 | 0.392 | 0.119 |
| 230 | Hebi | 0.318 | 0.067 | 0.120 | 0.357 | 0.119 |
| 231 | Bayanzhuoer | 0.296 | 0.061 | 0.146 | 0.387 | 0.118 |
| 232 | Bozhou | 0.352 | 0.054 | 0.105 | 0.445 | 0.118 |
| 233 | Baise | 0.308 | 0.070 | 0.118 | 0.336 | 0.118 |
| 234 | Shuangyashan | 0.221 | 0.076 | 0.153 | 0.332 | 0.116 |
| 235 | Heyuan | 0.323 | 0.055 | 0.150 | 0.352 | 0.115 |
| 236 | Tieling | 0.291 | 0.067 | 0.110 | 0.366 | 0.114 |
| 237 | Shuozhou | 0.317 | 0.058 | 0.138 | 0.356 | 0.114 |
| 238 | Lijiang | 0.284 | 0.059 | 0.204 | 0.254 | 0.113 |
| 239 | Neijiang | 0.286 | 0.068 | 0.088 | 0.398 | 0.113 |
| 240 | Dazhou | 0.307 | 0.064 | 0.110 | 0.354 | 0.113 |
| 241 | Zhangye | 0.262 | 0.063 | 0.192 | 0.271 | 0.113 |
| 242 | Wuwei | 0.278 | 0.071 | 0.148 | 0.256 | 0.112 |
| 243 | Ankang | 0.297 | 0.058 | 0.159 | 0.318 | 0.112 |
| 244 | Jixi | 0.232 | 0.068 | 0.143 | 0.358 | 0.112 |
| 245 | Meishan | 0.338 | 0.054 | 0.094 | 0.415 | 0.111 |
| 246 | Suining | 0.275 | 0.056 | 0.137 | 0.409 | 0.111 |
| 247 | Songyuan | 0.306 | 0.056 | 0.133 | 0.361 | 0.111 |
| 248 | Lvliang | 0.311 | 0.068 | 0.100 | 0.306 | 0.111 |
| 249 | Suizhou | 0.325 | 0.047 | 0.159 | 0.339 | 0.109 |
| 250 | Yichun | 0.222 | 0.067 | 0.152 | 0.338 | 0.109 |
| 251 | Chaoyang | 0.290 | 0.064 | 0.095 | 0.368 | 0.109 |
| 252 | Shanwei | 0.361 | 0.049 | 0.140 | 0.295 | 0.108 |
| 253 | Ziyang | 0.285 | 0.056 | 0.132 | 0.365 | 0.108 |
| 254 | Anshun | 0.327 | 0.054 | 0.111 | 0.357 | 0.108 |
| 255 | Tianshui | 0.265 | 0.063 | 0.115 | 0.356 | 0.107 |
| 256 | Fangchenggang | 0.279 | 0.052 | 0.156 | 0.341 | 0.106 |
| 257 | Wulanchabu | 0.287 | 0.053 | 0.136 | 0.350 | 0.105 |
| 258 | Yunfu | 0.340 | 0.059 | 0.111 | 0.262 | 0.105 |
| 259 | Wuzhong | 0.262 | 0.062 | 0.151 | 0.266 | 0.104 |
| 260 | Guigang | 0.303 | 0.052 | 0.097 | 0.396 | 0.103 |
| 261 | Liaoyuan | 0.261 | 0.062 | 0.133 | 0.296 | 0.103 |
| 262 | Hechi | 0.274 | 0.061 | 0.120 | 0.292 | 0.102 |
| 263 | Guangan | 0.321 | 0.047 | 0.118 | 0.340 | 0.101 |
| 264 | Zhangjiajie | 0.310 | 0.052 | 0.125 | 0.303 | 0.101 |
| 265 | Yingtan | 0.320 | 0.050 | 0.136 | 0.260 | 0.099 |
| 266 | Bazhong | 0.244 | 0.050 | 0.161 | 0.326 | 0.099 |
| 267 | Simao | 0.262 | 0.048 | 0.174 | 0.285 | 0.098 |
| 268 | Suihua | 0.273 | 0.054 | 0.112 | 0.330 | 0.098 |
| 269 | Laibin | 0.278 | 0.049 | 0.098 | 0.363 | 0.095 |
| 270 | Qitaihe | 0.207 | 0.057 | 0.149 | 0.292 | 0.094 |
| 271 | Chongzuo | 0.268 | 0.061 | 0.101 | 0.252 | 0.094 |
| 272 | Hezhou | 0.286 | 0.044 | 0.129 | 0.313 | 0.093 |
| 273 | Zhaotong | 0.262 | 0.063 | 0.086 | 0.267 | 0.093 |
| 274 | Heihe | 0.232 | 0.057 | 0.159 | 0.213 | 0.092 |
| 275 | Pingliang | 0.244 | 0.051 | 0.139 | 0.274 | 0.091 |
| 276 | Baoshan | 0.268 | 0.052 | 0.114 | 0.261 | 0.089 |
| 277 | Shangluo | 0.260 | 0.053 | 0.116 | 0.237 | 0.087 |
| 278 | Qingyang | 0.252 | 0.048 | 0.131 | 0.252 | 0.086 |
| 279 | Baiyin | 0.243 | 0.043 | 0.121 | 0.329 | 0.086 |
| 280 | Zhongwei | 0.221 | 0.042 | 0.155 | 0.283 | 0.084 |
| 281 | Guyuan | 0.211 | 0.053 | 0.123 | 0.251 | 0.082 |
| 282 | Lincang | 0.245 | 0.042 | 0.128 | 0.219 | 0.077 |
| 283 | Dingxi | 0.242 | 0.044 | 0.097 | 0.163 | 0.067 |
| 284 | Longnan | 0.215 | 0.050 | 0.099 | 0.104 | 0.063 |

S2 Table. ECO, SCO, ENV and INF’s performances of total and three major divisions 2011-2019

| Name | Area | Number of Cities | Average value | Standard Deviation | Coefficient of variation | Median |
| --- | --- | --- | --- | --- | --- | --- |
| ECO | National | 284 | 0.387 | 0.102 | 0.264 | 0.370 |
|  | East | 114 | 0.437 | 0.110 | 0.253 | 0.418 |
|  | Central | 60 | 0.369 | 0.076 | 0.207 | 0.360 |
|  | West | 109 | 0.329 | 0.084 | 0.255 | 0.321 |
| SCO | National | 284 | 0.128 | 0.110 | 0.860 | 0.087 |
|  | East | 114 | 0.156 | 0.130 | 0.835 | 0.109 |
|  | Central | 61 | 0.107 | 0.074 | 0.695 | 0.083 |
|  | West | 109 | 0.111 | 0.111 | 1.000 | 0.070 |
| ENV | National | 284 | 0.141 | 0.060 | 0.426 | 0.132 |
|  | East | 114 | 0.146 | 0.064 | 0.440 | 0.137 |
|  | Central | 61 | 0.130 | 0.033 | 0.253 | 0.126 |
|  | West | 109 | 0.151 | 0.083 | 0.551 | 0.132 |
| INF | National | 284 | 0.465 | 0.141 | 0.302 | 0.446 |
|  | East | 114 | 0.531 | 0.144 | 0.270 | 0.520 |
|  | Central | 61 | 0.442 | 0.100 | 0.227 | 0.433 |
|  | West | 109 | 0.384 | 0.142 | 0.370 | 0.357 |

S3 Table. Mean values of barrier degree for each factor, 2011-2019

| code | X1 | X2 | X3 | X4 | X5 | X6 | X7 | X8 | X9 | X10 | X11 | X12 |
| --- | --- | --- | --- | --- | --- | --- | --- | --- | --- | --- | --- | --- |
| 110000 | 0.012 | 0.009 | 0.026 | 0.023 | 0.048 | 0.099 | 0.336 | 0.089 | 0.027 | 0.290 | 0.032 | 0.008 |
| 120000 | 0.013 | 0.011 | 0.022 | 0.049 | 0.069 | 0.164 | 0.241 | 0.164 | 0.029 | 0.204 | 0.021 | 0.011 |
| 130100 | 0.016 | 0.013 | 0.020 | 0.047 | 0.068 | 0.152 | 0.261 | 0.166 | 0.030 | 0.178 | 0.033 | 0.015 |
| 130200 | 0.015 | 0.015 | 0.019 | 0.046 | 0.064 | 0.218 | 0.231 | 0.160 | 0.024 | 0.161 | 0.035 | 0.013 |
| 130300 | 0.014 | 0.020 | 0.021 | 0.048 | 0.065 | 0.206 | 0.229 | 0.166 | 0.016 | 0.155 | 0.040 | 0.018 |
| 130400 | 0.017 | 0.014 | 0.021 | 0.045 | 0.067 | 0.212 | 0.232 | 0.160 | 0.026 | 0.155 | 0.036 | 0.018 |
| 130500 | 0.017 | 0.016 | 0.021 | 0.044 | 0.066 | 0.213 | 0.224 | 0.159 | 0.029 | 0.152 | 0.041 | 0.018 |
| 130600 | 0.018 | 0.013 | 0.020 | 0.046 | 0.067 | 0.198 | 0.239 | 0.160 | 0.026 | 0.159 | 0.036 | 0.018 |
| 130700 | 0.016 | 0.019 | 0.021 | 0.047 | 0.067 | 0.221 | 0.221 | 0.162 | 0.014 | 0.151 | 0.042 | 0.019 |
| 130800 | 0.016 | 0.020 | 0.022 | 0.047 | 0.065 | 0.218 | 0.223 | 0.163 | 0.013 | 0.147 | 0.046 | 0.020 |
| 130900 | 0.016 | 0.016 | 0.020 | 0.044 | 0.064 | 0.211 | 0.228 | 0.159 | 0.028 | 0.152 | 0.043 | 0.019 |
| 131000 | 0.015 | 0.017 | 0.019 | 0.043 | 0.069 | 0.202 | 0.230 | 0.163 | 0.027 | 0.153 | 0.043 | 0.019 |
| 131100 | 0.017 | 0.019 | 0.021 | 0.044 | 0.067 | 0.219 | 0.219 | 0.158 | 0.030 | 0.142 | 0.044 | 0.020 |
| 140100 | 0.015 | 0.017 | 0.024 | 0.045 | 0.058 | 0.133 | 0.265 | 0.175 | 0.020 | 0.190 | 0.040 | 0.016 |
| 140200 | 0.015 | 0.019 | 0.020 | 0.049 | 0.065 | 0.227 | 0.218 | 0.161 | 0.015 | 0.153 | 0.041 | 0.018 |
| 140300 | 0.014 | 0.022 | 0.022 | 0.049 | 0.064 | 0.223 | 0.211 | 0.161 | 0.020 | 0.146 | 0.049 | 0.018 |
| 140400 | 0.016 | 0.018 | 0.021 | 0.048 | 0.065 | 0.217 | 0.219 | 0.160 | 0.019 | 0.151 | 0.046 | 0.019 |
| 140500 | 0.015 | 0.020 | 0.020 | 0.048 | 0.065 | 0.225 | 0.213 | 0.159 | 0.019 | 0.147 | 0.049 | 0.020 |
| 140600 | 0.014 | 0.022 | 0.020 | 0.049 | 0.069 | 0.222 | 0.214 | 0.161 | 0.017 | 0.143 | 0.048 | 0.021 |
| 140700 | 0.017 | 0.019 | 0.021 | 0.048 | 0.069 | 0.193 | 0.227 | 0.164 | 0.022 | 0.154 | 0.046 | 0.021 |
| 140800 | 0.017 | 0.018 | 0.022 | 0.048 | 0.061 | 0.212 | 0.221 | 0.159 | 0.025 | 0.150 | 0.048 | 0.021 |
| 140900 | 0.017 | 0.020 | 0.021 | 0.050 | 0.059 | 0.217 | 0.216 | 0.161 | 0.021 | 0.149 | 0.046 | 0.022 |
| 141000 | 0.016 | 0.018 | 0.021 | 0.048 | 0.065 | 0.215 | 0.221 | 0.159 | 0.021 | 0.147 | 0.049 | 0.020 |
| 141100 | 0.017 | 0.018 | 0.022 | 0.048 | 0.062 | 0.219 | 0.217 | 0.158 | 0.020 | 0.147 | 0.049 | 0.024 |
| 150100 | 0.010 | 0.020 | 0.023 | 0.044 | 0.070 | 0.171 | 0.251 | 0.166 | 0.015 | 0.167 | 0.043 | 0.018 |
| 150200 | 0.011 | 0.021 | 0.020 | 0.044 | 0.064 | 0.223 | 0.227 | 0.165 | 0.013 | 0.157 | 0.042 | 0.014 |
| 150300 | 0.014 | 0.030 | 0.024 | 0.048 | 0.064 | 0.236 | 0.215 | 0.164 | 0.019 | 0.116 | 0.051 | 0.020 |
| 150400 | 0.016 | 0.019 | 0.023 | 0.046 | 0.064 | 0.222 | 0.224 | 0.162 | 0.012 | 0.151 | 0.041 | 0.020 |
| 150500 | 0.016 | 0.021 | 0.023 | 0.046 | 0.065 | 0.220 | 0.220 | 0.161 | 0.015 | 0.147 | 0.045 | 0.021 |
| 150600 | 0.010 | 0.023 | 0.020 | 0.045 | 0.064 | 0.237 | 0.227 | 0.158 | 0.011 | 0.145 | 0.040 | 0.021 |
| 150700 | 0.015 | 0.021 | 0.023 | 0.048 | 0.062 | 0.225 | 0.212 | 0.159 | 0.014 | 0.147 | 0.050 | 0.024 |
| 150800 | 0.016 | 0.024 | 0.022 | 0.046 | 0.065 | 0.224 | 0.216 | 0.162 | 0.016 | 0.143 | 0.045 | 0.021 |
| 150900 | 0.015 | 0.023 | 0.023 | 0.047 | 0.069 | 0.220 | 0.216 | 0.160 | 0.012 | 0.145 | 0.048 | 0.021 |
| 210100 | 0.014 | 0.015 | 0.022 | 0.045 | 0.062 | 0.164 | 0.250 | 0.172 | 0.021 | 0.189 | 0.032 | 0.014 |
| 210200 | 0.013 | 0.016 | 0.020 | 0.044 | 0.065 | 0.189 | 0.245 | 0.166 | 0.016 | 0.179 | 0.034 | 0.013 |
| 210300 | 0.013 | 0.019 | 0.021 | 0.046 | 0.066 | 0.229 | 0.216 | 0.160 | 0.017 | 0.154 | 0.041 | 0.016 |
| 210400 | 0.015 | 0.023 | 0.023 | 0.048 | 0.066 | 0.224 | 0.211 | 0.165 | 0.015 | 0.152 | 0.044 | 0.015 |
| 210500 | 0.015 | 0.024 | 0.024 | 0.051 | 0.057 | 0.219 | 0.210 | 0.168 | 0.012 | 0.152 | 0.049 | 0.019 |
| 210600 | 0.015 | 0.022 | 0.022 | 0.047 | 0.062 | 0.225 | 0.213 | 0.163 | 0.016 | 0.149 | 0.045 | 0.019 |
| 210700 | 0.016 | 0.021 | 0.023 | 0.046 | 0.065 | 0.208 | 0.217 | 0.163 | 0.019 | 0.156 | 0.046 | 0.022 |
| 210800 | 0.014 | 0.022 | 0.022 | 0.044 | 0.067 | 0.226 | 0.214 | 0.161 | 0.016 | 0.151 | 0.044 | 0.018 |
| 210900 | 0.017 | 0.023 | 0.025 | 0.048 | 0.065 | 0.216 | 0.211 | 0.161 | 0.017 | 0.149 | 0.049 | 0.020 |
| 211000 | 0.015 | 0.024 | 0.023 | 0.049 | 0.064 | 0.225 | 0.211 | 0.161 | 0.019 | 0.148 | 0.043 | 0.018 |
| 211100 | 0.015 | 0.022 | 0.021 | 0.051 | 0.059 | 0.235 | 0.208 | 0.166 | 0.019 | 0.141 | 0.043 | 0.020 |
| 211200 | 0.016 | 0.022 | 0.024 | 0.046 | 0.067 | 0.217 | 0.213 | 0.161 | 0.018 | 0.146 | 0.046 | 0.022 |
| 211300 | 0.016 | 0.021 | 0.024 | 0.047 | 0.066 | 0.222 | 0.210 | 0.160 | 0.019 | 0.148 | 0.046 | 0.021 |
| 211400 | 0.016 | 0.022 | 0.023 | 0.048 | 0.061 | 0.225 | 0.214 | 0.161 | 0.016 | 0.149 | 0.046 | 0.018 |
| 220100 | 0.016 | 0.015 | 0.022 | 0.046 | 0.066 | 0.167 | 0.255 | 0.170 | 0.020 | 0.177 | 0.031 | 0.015 |
| 220200 | 0.014 | 0.019 | 0.022 | 0.045 | 0.063 | 0.216 | 0.225 | 0.164 | 0.016 | 0.159 | 0.042 | 0.015 |
| 220300 | 0.017 | 0.021 | 0.023 | 0.046 | 0.063 | 0.215 | 0.216 | 0.160 | 0.020 | 0.149 | 0.047 | 0.021 |
| 220400 | 0.015 | 0.026 | 0.023 | 0.047 | 0.064 | 0.222 | 0.210 | 0.159 | 0.018 | 0.141 | 0.050 | 0.023 |
| 220500 | 0.015 | 0.023 | 0.022 | 0.044 | 0.064 | 0.226 | 0.210 | 0.161 | 0.017 | 0.145 | 0.050 | 0.022 |
| 220600 | 0.015 | 0.026 | 0.022 | 0.047 | 0.061 | 0.228 | 0.210 | 0.159 | 0.017 | 0.141 | 0.051 | 0.024 |
| 220700 | 0.014 | 0.022 | 0.022 | 0.048 | 0.068 | 0.224 | 0.215 | 0.159 | 0.017 | 0.143 | 0.048 | 0.020 |
| 220800 | 0.016 | 0.023 | 0.024 | 0.048 | 0.065 | 0.220 | 0.211 | 0.162 | 0.018 | 0.140 | 0.050 | 0.022 |
| 230100 | 0.015 | 0.014 | 0.021 | 0.048 | 0.067 | 0.152 | 0.265 | 0.168 | 0.019 | 0.181 | 0.035 | 0.016 |
| 230200 | 0.017 | 0.019 | 0.023 | 0.048 | 0.058 | 0.220 | 0.225 | 0.163 | 0.013 | 0.153 | 0.043 | 0.017 |
| 230300 | 0.017 | 0.023 | 0.023 | 0.052 | 0.065 | 0.225 | 0.210 | 0.158 | 0.013 | 0.144 | 0.047 | 0.022 |
| 230400 | 0.018 | 0.026 | 0.025 | 0.052 | 0.060 | 0.226 | 0.209 | 0.160 | 0.013 | 0.138 | 0.051 | 0.022 |
| 230500 | 0.018 | 0.025 | 0.025 | 0.050 | 0.063 | 0.220 | 0.214 | 0.158 | 0.013 | 0.142 | 0.050 | 0.021 |
| 230600 | 0.014 | 0.020 | 0.021 | 0.042 | 0.063 | 0.231 | 0.208 | 0.169 | 0.012 | 0.163 | 0.039 | 0.017 |
| 230700 | 0.020 | 0.026 | 0.021 | 0.050 | 0.065 | 0.227 | 0.209 | 0.157 | 0.014 | 0.141 | 0.046 | 0.024 |
| 230800 | 0.016 | 0.022 | 0.023 | 0.048 | 0.066 | 0.217 | 0.217 | 0.162 | 0.013 | 0.147 | 0.047 | 0.021 |
| 230900 | 0.016 | 0.027 | 0.025 | 0.048 | 0.066 | 0.222 | 0.208 | 0.158 | 0.016 | 0.139 | 0.052 | 0.021 |
| 231000 | 0.015 | 0.022 | 0.020 | 0.047 | 0.064 | 0.215 | 0.222 | 0.163 | 0.015 | 0.149 | 0.045 | 0.023 |
| 231100 | 0.018 | 0.023 | 0.023 | 0.050 | 0.061 | 0.221 | 0.213 | 0.158 | 0.015 | 0.138 | 0.056 | 0.024 |
| 232300 | 0.019 | 0.020 | 0.023 | 0.047 | 0.064 | 0.221 | 0.216 | 0.157 | 0.019 | 0.144 | 0.049 | 0.022 |
| 310000 | 0.012 | 0.009 | 0.020 | 0.047 | 0.059 | 0.170 | 0.276 | 0.117 | 0.015 | 0.246 | 0.025 | 0.006 |
| 320100 | 0.012 | 0.013 | 0.020 | 0.042 | 0.072 | 0.118 | 0.284 | 0.171 | 0.018 | 0.209 | 0.027 | 0.014 |
| 320200 | 0.011 | 0.016 | 0.018 | 0.039 | 0.062 | 0.234 | 0.228 | 0.165 | 0.018 | 0.171 | 0.025 | 0.013 |
| 320300 | 0.014 | 0.013 | 0.018 | 0.042 | 0.066 | 0.213 | 0.240 | 0.164 | 0.022 | 0.160 | 0.033 | 0.014 |
| 320400 | 0.011 | 0.018 | 0.017 | 0.037 | 0.061 | 0.227 | 0.232 | 0.165 | 0.020 | 0.166 | 0.030 | 0.014 |
| 320500 | 0.012 | 0.013 | 0.018 | 0.041 | 0.061 | 0.224 | 0.233 | 0.155 | 0.019 | 0.189 | 0.023 | 0.013 |
| 320600 | 0.013 | 0.014 | 0.017 | 0.042 | 0.068 | 0.230 | 0.234 | 0.163 | 0.017 | 0.158 | 0.029 | 0.016 |
| 320700 | 0.016 | 0.017 | 0.019 | 0.042 | 0.069 | 0.223 | 0.227 | 0.164 | 0.017 | 0.154 | 0.036 | 0.018 |
| 320800 | 0.015 | 0.017 | 0.020 | 0.042 | 0.066 | 0.222 | 0.229 | 0.166 | 0.019 | 0.154 | 0.034 | 0.016 |
| 320900 | 0.015 | 0.015 | 0.019 | 0.040 | 0.067 | 0.226 | 0.231 | 0.163 | 0.018 | 0.154 | 0.036 | 0.017 |
| 321000 | 0.013 | 0.016 | 0.018 | 0.040 | 0.068 | 0.224 | 0.229 | 0.165 | 0.019 | 0.155 | 0.034 | 0.017 |
| 321100 | 0.012 | 0.020 | 0.019 | 0.039 | 0.066 | 0.220 | 0.229 | 0.168 | 0.020 | 0.154 | 0.037 | 0.017 |
| 321200 | 0.014 | 0.016 | 0.018 | 0.042 | 0.066 | 0.228 | 0.224 | 0.166 | 0.021 | 0.154 | 0.036 | 0.016 |
| 321300 | 0.016 | 0.017 | 0.020 | 0.041 | 0.070 | 0.222 | 0.227 | 0.164 | 0.019 | 0.150 | 0.037 | 0.017 |
| 330100 | 0.011 | 0.012 | 0.018 | 0.040 | 0.056 | 0.183 | 0.256 | 0.159 | 0.016 | 0.206 | 0.029 | 0.014 |
| 330200 | 0.012 | 0.014 | 0.018 | 0.041 | 0.062 | 0.232 | 0.227 | 0.157 | 0.015 | 0.176 | 0.032 | 0.013 |
| 330300 | 0.013 | 0.014 | 0.018 | 0.042 | 0.064 | 0.227 | 0.240 | 0.154 | 0.015 | 0.163 | 0.032 | 0.017 |
| 330400 | 0.013 | 0.017 | 0.018 | 0.040 | 0.061 | 0.233 | 0.216 | 0.160 | 0.020 | 0.162 | 0.044 | 0.016 |
| 330500 | 0.013 | 0.020 | 0.019 | 0.040 | 0.065 | 0.235 | 0.217 | 0.164 | 0.017 | 0.153 | 0.038 | 0.018 |
| 330600 | 0.012 | 0.015 | 0.019 | 0.040 | 0.064 | 0.228 | 0.224 | 0.164 | 0.017 | 0.165 | 0.037 | 0.015 |
| 330700 | 0.012 | 0.016 | 0.019 | 0.039 | 0.063 | 0.228 | 0.229 | 0.158 | 0.018 | 0.160 | 0.038 | 0.020 |
| 330800 | 0.013 | 0.022 | 0.021 | 0.040 | 0.067 | 0.231 | 0.217 | 0.161 | 0.016 | 0.151 | 0.042 | 0.018 |
| 330900 | 0.011 | 0.025 | 0.020 | 0.047 | 0.062 | 0.234 | 0.217 | 0.160 | 0.012 | 0.144 | 0.047 | 0.022 |
| 331000 | 0.012 | 0.015 | 0.019 | 0.042 | 0.066 | 0.232 | 0.229 | 0.160 | 0.014 | 0.157 | 0.037 | 0.016 |
| 331100 | 0.013 | 0.022 | 0.020 | 0.042 | 0.064 | 0.229 | 0.221 | 0.164 | 0.015 | 0.143 | 0.045 | 0.020 |
| 340100 | 0.015 | 0.013 | 0.019 | 0.039 | 0.070 | 0.151 | 0.266 | 0.178 | 0.021 | 0.183 | 0.029 | 0.017 |
| 340200 | 0.015 | 0.018 | 0.019 | 0.032 | 0.069 | 0.213 | 0.232 | 0.170 | 0.020 | 0.160 | 0.034 | 0.017 |
| 340300 | 0.016 | 0.019 | 0.020 | 0.041 | 0.068 | 0.218 | 0.224 | 0.165 | 0.021 | 0.150 | 0.040 | 0.019 |
| 340400 | 0.016 | 0.019 | 0.021 | 0.046 | 0.067 | 0.215 | 0.221 | 0.165 | 0.020 | 0.149 | 0.042 | 0.019 |
| 340500 | 0.014 | 0.022 | 0.019 | 0.040 | 0.071 | 0.217 | 0.221 | 0.167 | 0.019 | 0.153 | 0.042 | 0.017 |
| 340600 | 0.017 | 0.020 | 0.021 | 0.045 | 0.067 | 0.216 | 0.215 | 0.163 | 0.021 | 0.151 | 0.045 | 0.020 |
| 340700 | 0.015 | 0.025 | 0.021 | 0.041 | 0.064 | 0.221 | 0.215 | 0.165 | 0.018 | 0.148 | 0.046 | 0.021 |
| 340800 | 0.017 | 0.018 | 0.020 | 0.042 | 0.069 | 0.220 | 0.225 | 0.163 | 0.018 | 0.151 | 0.040 | 0.018 |
| 341000 | 0.015 | 0.025 | 0.021 | 0.042 | 0.067 | 0.226 | 0.221 | 0.163 | 0.011 | 0.139 | 0.047 | 0.023 |
| 341100 | 0.018 | 0.019 | 0.020 | 0.040 | 0.070 | 0.219 | 0.223 | 0.164 | 0.019 | 0.150 | 0.039 | 0.020 |
| 341200 | 0.019 | 0.016 | 0.021 | 0.043 | 0.068 | 0.212 | 0.228 | 0.161 | 0.022 | 0.152 | 0.038 | 0.019 |
| 341300 | 0.018 | 0.016 | 0.021 | 0.046 | 0.068 | 0.217 | 0.222 | 0.158 | 0.022 | 0.151 | 0.040 | 0.021 |
| 341500 | 0.020 | 0.015 | 0.022 | 0.048 | 0.068 | 0.234 | 0.180 | 0.169 | 0.019 | 0.157 | 0.045 | 0.023 |
| 341600 | 0.018 | 0.019 | 0.022 | 0.042 | 0.071 | 0.216 | 0.221 | 0.161 | 0.022 | 0.146 | 0.039 | 0.021 |
| 341700 | 0.016 | 0.025 | 0.021 | 0.042 | 0.070 | 0.218 | 0.219 | 0.161 | 0.017 | 0.145 | 0.045 | 0.021 |
| 341800 | 0.016 | 0.021 | 0.020 | 0.040 | 0.068 | 0.229 | 0.221 | 0.157 | 0.017 | 0.149 | 0.042 | 0.020 |
| 350100 | 0.014 | 0.012 | 0.019 | 0.046 | 0.069 | 0.179 | 0.256 | 0.165 | 0.012 | 0.177 | 0.035 | 0.017 |
| 350200 | 0.013 | 0.017 | 0.021 | 0.046 | 0.060 | 0.215 | 0.195 | 0.171 | 0.014 | 0.193 | 0.035 | 0.019 |
| 350300 | 0.016 | 0.017 | 0.020 | 0.044 | 0.069 | 0.228 | 0.227 | 0.161 | 0.013 | 0.148 | 0.040 | 0.018 |
| 350400 | 0.014 | 0.020 | 0.020 | 0.043 | 0.067 | 0.222 | 0.219 | 0.161 | 0.015 | 0.147 | 0.050 | 0.021 |
| 350500 | 0.015 | 0.012 | 0.019 | 0.045 | 0.072 | 0.206 | 0.243 | 0.160 | 0.012 | 0.164 | 0.035 | 0.018 |
| 350600 | 0.016 | 0.016 | 0.019 | 0.044 | 0.069 | 0.217 | 0.228 | 0.163 | 0.015 | 0.153 | 0.042 | 0.019 |
| 350700 | 0.016 | 0.020 | 0.020 | 0.043 | 0.066 | 0.222 | 0.219 | 0.161 | 0.015 | 0.145 | 0.052 | 0.022 |
| 350800 | 0.014 | 0.019 | 0.019 | 0.044 | 0.063 | 0.227 | 0.221 | 0.162 | 0.014 | 0.149 | 0.047 | 0.020 |
| 350900 | 0.016 | 0.020 | 0.020 | 0.042 | 0.068 | 0.224 | 0.219 | 0.161 | 0.015 | 0.146 | 0.048 | 0.023 |
| 360100 | 0.016 | 0.014 | 0.021 | 0.049 | 0.075 | 0.114 | 0.271 | 0.182 | 0.018 | 0.182 | 0.040 | 0.019 |
| 360200 | 0.016 | 0.023 | 0.021 | 0.047 | 0.066 | 0.219 | 0.218 | 0.164 | 0.013 | 0.150 | 0.045 | 0.019 |
| 360300 | 0.015 | 0.022 | 0.021 | 0.044 | 0.065 | 0.221 | 0.216 | 0.163 | 0.018 | 0.147 | 0.050 | 0.018 |
| 360400 | 0.017 | 0.017 | 0.020 | 0.043 | 0.067 | 0.213 | 0.228 | 0.165 | 0.015 | 0.154 | 0.041 | 0.020 |
| 360500 | 0.014 | 0.024 | 0.021 | 0.045 | 0.067 | 0.212 | 0.220 | 0.166 | 0.015 | 0.149 | 0.044 | 0.021 |
| 360600 | 0.015 | 0.023 | 0.021 | 0.043 | 0.068 | 0.220 | 0.213 | 0.161 | 0.017 | 0.141 | 0.052 | 0.024 |
| 360700 | 0.018 | 0.015 | 0.020 | 0.044 | 0.066 | 0.208 | 0.235 | 0.163 | 0.014 | 0.158 | 0.039 | 0.020 |
| 360800 | 0.018 | 0.018 | 0.020 | 0.044 | 0.068 | 0.221 | 0.223 | 0.162 | 0.016 | 0.147 | 0.042 | 0.022 |
| 360900 | 0.018 | 0.017 | 0.021 | 0.042 | 0.067 | 0.218 | 0.224 | 0.164 | 0.017 | 0.150 | 0.042 | 0.020 |
| 361000 | 0.018 | 0.018 | 0.021 | 0.044 | 0.071 | 0.218 | 0.222 | 0.164 | 0.014 | 0.148 | 0.041 | 0.020 |
| 361100 | 0.018 | 0.016 | 0.021 | 0.044 | 0.069 | 0.217 | 0.225 | 0.163 | 0.015 | 0.150 | 0.041 | 0.020 |
| 370100 | 0.013 | 0.014 | 0.022 | 0.049 | 0.065 | 0.109 | 0.277 | 0.175 | 0.028 | 0.196 | 0.034 | 0.017 |
| 370200 | 0.012 | 0.013 | 0.019 | 0.044 | 0.066 | 0.204 | 0.248 | 0.161 | 0.017 | 0.171 | 0.029 | 0.015 |
| 370300 | 0.014 | 0.016 | 0.020 | 0.045 | 0.063 | 0.224 | 0.228 | 0.165 | 0.020 | 0.157 | 0.034 | 0.014 |
| 370400 | 0.015 | 0.018 | 0.021 | 0.045 | 0.065 | 0.222 | 0.221 | 0.162 | 0.023 | 0.152 | 0.038 | 0.018 |
| 370500 | 0.012 | 0.020 | 0.019 | 0.044 | 0.066 | 0.227 | 0.222 | 0.165 | 0.020 | 0.149 | 0.040 | 0.016 |
| 370600 | 0.014 | 0.015 | 0.019 | 0.042 | 0.064 | 0.222 | 0.233 | 0.164 | 0.015 | 0.161 | 0.035 | 0.016 |
| 370700 | 0.016 | 0.014 | 0.020 | 0.042 | 0.062 | 0.211 | 0.239 | 0.159 | 0.021 | 0.163 | 0.037 | 0.017 |
| 370800 | 0.015 | 0.014 | 0.020 | 0.045 | 0.063 | 0.215 | 0.230 | 0.161 | 0.025 | 0.157 | 0.036 | 0.018 |
| 370900 | 0.015 | 0.016 | 0.021 | 0.045 | 0.065 | 0.215 | 0.224 | 0.163 | 0.024 | 0.155 | 0.038 | 0.019 |
| 371000 | 0.013 | 0.020 | 0.019 | 0.043 | 0.062 | 0.226 | 0.226 | 0.170 | 0.013 | 0.151 | 0.037 | 0.019 |
| 371100 | 0.015 | 0.020 | 0.020 | 0.045 | 0.067 | 0.224 | 0.220 | 0.163 | 0.020 | 0.151 | 0.039 | 0.017 |
| 371300 | 0.016 | 0.019 | 0.021 | 0.045 | 0.066 | 0.219 | 0.232 | 0.158 | 0.021 | 0.158 | 0.032 | 0.014 |
| 371400 | 0.016 | 0.015 | 0.021 | 0.043 | 0.067 | 0.220 | 0.223 | 0.163 | 0.025 | 0.152 | 0.038 | 0.018 |
| 371500 | 0.016 | 0.016 | 0.022 | 0.045 | 0.066 | 0.218 | 0.223 | 0.160 | 0.026 | 0.152 | 0.038 | 0.018 |
| 371600 | 0.015 | 0.018 | 0.021 | 0.044 | 0.066 | 0.222 | 0.222 | 0.163 | 0.022 | 0.154 | 0.039 | 0.016 |
| 371700 | 0.018 | 0.015 | 0.023 | 0.045 | 0.065 | 0.210 | 0.227 | 0.160 | 0.025 | 0.154 | 0.038 | 0.019 |
| 410100 | 0.016 | 0.018 | 0.020 | 0.050 | 0.061 | 0.105 | 0.284 | 0.173 | 0.030 | 0.197 | 0.033 | 0.015 |
| 410200 | 0.017 | 0.011 | 0.021 | 0.046 | 0.065 | 0.211 | 0.225 | 0.163 | 0.026 | 0.152 | 0.044 | 0.018 |
| 410300 | 0.015 | 0.016 | 0.019 | 0.041 | 0.064 | 0.218 | 0.234 | 0.162 | 0.019 | 0.159 | 0.039 | 0.015 |
| 410400 | 0.017 | 0.015 | 0.021 | 0.047 | 0.064 | 0.212 | 0.221 | 0.163 | 0.024 | 0.150 | 0.044 | 0.022 |
| 410500 | 0.017 | 0.016 | 0.020 | 0.046 | 0.065 | 0.211 | 0.226 | 0.162 | 0.027 | 0.153 | 0.042 | 0.015 |
| 410600 | 0.017 | 0.018 | 0.022 | 0.047 | 0.066 | 0.217 | 0.216 | 0.161 | 0.026 | 0.142 | 0.047 | 0.021 |
| 410700 | 0.018 | 0.019 | 0.020 | 0.044 | 0.066 | 0.201 | 0.226 | 0.163 | 0.026 | 0.155 | 0.042 | 0.020 |
| 410800 | 0.016 | 0.017 | 0.020 | 0.045 | 0.066 | 0.211 | 0.223 | 0.164 | 0.027 | 0.151 | 0.043 | 0.018 |
| 410900 | 0.016 | 0.017 | 0.020 | 0.046 | 0.066 | 0.218 | 0.218 | 0.159 | 0.027 | 0.146 | 0.044 | 0.021 |
| 411000 | 0.017 | 0.018 | 0.020 | 0.044 | 0.067 | 0.215 | 0.221 | 0.161 | 0.026 | 0.147 | 0.044 | 0.020 |
| 411100 | 0.018 | 0.019 | 0.020 | 0.048 | 0.066 | 0.216 | 0.218 | 0.162 | 0.026 | 0.144 | 0.046 | 0.019 |
| 411200 | 0.016 | 0.020 | 0.019 | 0.047 | 0.064 | 0.223 | 0.216 | 0.159 | 0.019 | 0.148 | 0.049 | 0.020 |
| 411300 | 0.018 | 0.018 | 0.020 | 0.046 | 0.066 | 0.206 | 0.234 | 0.164 | 0.022 | 0.151 | 0.038 | 0.018 |
| 411400 | 0.019 | 0.013 | 0.021 | 0.047 | 0.065 | 0.206 | 0.228 | 0.161 | 0.025 | 0.153 | 0.043 | 0.018 |
| 411500 | 0.018 | 0.015 | 0.020 | 0.047 | 0.071 | 0.209 | 0.226 | 0.162 | 0.020 | 0.147 | 0.046 | 0.019 |
| 411600 | 0.020 | 0.015 | 0.021 | 0.046 | 0.069 | 0.207 | 0.227 | 0.161 | 0.024 | 0.149 | 0.043 | 0.019 |
| 411700 | 0.019 | 0.015 | 0.021 | 0.047 | 0.066 | 0.211 | 0.226 | 0.162 | 0.022 | 0.149 | 0.042 | 0.020 |
| 420100 | 0.016 | 0.019 | 0.021 | 0.047 | 0.068 | 0.063 | 0.304 | 0.174 | 0.027 | 0.217 | 0.030 | 0.015 |
| 420200 | 0.016 | 0.013 | 0.021 | 0.045 | 0.064 | 0.223 | 0.221 | 0.166 | 0.021 | 0.153 | 0.041 | 0.017 |
| 420300 | 0.017 | 0.020 | 0.021 | 0.047 | 0.059 | 0.223 | 0.221 | 0.165 | 0.015 | 0.147 | 0.044 | 0.020 |
| 420500 | 0.016 | 0.018 | 0.020 | 0.040 | 0.064 | 0.229 | 0.221 | 0.161 | 0.018 | 0.154 | 0.041 | 0.019 |
| 420600 | 0.017 | 0.016 | 0.019 | 0.042 | 0.063 | 0.226 | 0.224 | 0.163 | 0.021 | 0.151 | 0.041 | 0.018 |
| 420700 | 0.016 | 0.018 | 0.021 | 0.049 | 0.064 | 0.226 | 0.212 | 0.163 | 0.024 | 0.142 | 0.045 | 0.020 |
| 420800 | 0.017 | 0.022 | 0.020 | 0.045 | 0.063 | 0.227 | 0.217 | 0.162 | 0.023 | 0.143 | 0.043 | 0.019 |
| 420900 | 0.018 | 0.018 | 0.020 | 0.044 | 0.065 | 0.222 | 0.217 | 0.161 | 0.024 | 0.148 | 0.042 | 0.021 |
| 421000 | 0.019 | 0.015 | 0.020 | 0.042 | 0.064 | 0.214 | 0.224 | 0.162 | 0.024 | 0.154 | 0.044 | 0.019 |
| 421100 | 0.019 | 0.017 | 0.020 | 0.047 | 0.062 | 0.216 | 0.223 | 0.161 | 0.022 | 0.146 | 0.046 | 0.023 |
| 421200 | 0.017 | 0.017 | 0.021 | 0.045 | 0.063 | 0.221 | 0.223 | 0.163 | 0.018 | 0.143 | 0.049 | 0.020 |
| 421300 | 0.017 | 0.022 | 0.020 | 0.044 | 0.068 | 0.224 | 0.218 | 0.160 | 0.018 | 0.139 | 0.049 | 0.021 |
| 430100 | 0.014 | 0.024 | 0.020 | 0.046 | 0.062 | 0.136 | 0.275 | 0.170 | 0.022 | 0.179 | 0.036 | 0.016 |
| 430200 | 0.015 | 0.014 | 0.020 | 0.040 | 0.067 | 0.217 | 0.230 | 0.168 | 0.017 | 0.155 | 0.039 | 0.018 |
| 430300 | 0.015 | 0.019 | 0.020 | 0.047 | 0.065 | 0.201 | 0.228 | 0.167 | 0.021 | 0.156 | 0.043 | 0.016 |
| 430400 | 0.017 | 0.017 | 0.020 | 0.046 | 0.065 | 0.209 | 0.229 | 0.165 | 0.020 | 0.155 | 0.040 | 0.017 |
| 430500 | 0.018 | 0.016 | 0.021 | 0.047 | 0.062 | 0.217 | 0.225 | 0.163 | 0.019 | 0.149 | 0.041 | 0.022 |
| 430600 | 0.015 | 0.018 | 0.020 | 0.044 | 0.067 | 0.222 | 0.223 | 0.164 | 0.019 | 0.150 | 0.040 | 0.019 |
| 430700 | 0.015 | 0.017 | 0.020 | 0.043 | 0.063 | 0.224 | 0.227 | 0.163 | 0.019 | 0.148 | 0.041 | 0.019 |
| 430800 | 0.013 | 0.020 | 0.023 | 0.048 | 0.067 | 0.218 | 0.216 | 0.161 | 0.018 | 0.142 | 0.050 | 0.023 |
| 430900 | 0.017 | 0.023 | 0.021 | 0.045 | 0.065 | 0.219 | 0.221 | 0.162 | 0.019 | 0.146 | 0.042 | 0.020 |
| 431000 | 0.016 | 0.020 | 0.019 | 0.044 | 0.065 | 0.221 | 0.225 | 0.162 | 0.017 | 0.147 | 0.044 | 0.020 |
| 431100 | 0.017 | 0.018 | 0.021 | 0.048 | 0.066 | 0.216 | 0.221 | 0.162 | 0.019 | 0.148 | 0.045 | 0.021 |
| 431200 | 0.016 | 0.018 | 0.022 | 0.046 | 0.062 | 0.219 | 0.222 | 0.162 | 0.018 | 0.148 | 0.047 | 0.020 |
| 431300 | 0.017 | 0.019 | 0.021 | 0.047 | 0.066 | 0.216 | 0.218 | 0.162 | 0.019 | 0.149 | 0.047 | 0.019 |
| 440100 | 0.011 | 0.027 | 0.025 | 0.049 | 0.062 | 0.059 | 0.314 | 0.147 | 0.013 | 0.257 | 0.026 | 0.010 |
| 440200 | 0.014 | 0.011 | 0.021 | 0.047 | 0.067 | 0.227 | 0.222 | 0.163 | 0.014 | 0.151 | 0.046 | 0.018 |
| 440300 | 0.012 | 0.033 | 0.027 | 0.038 | 0.053 | 0.381 | 0.095 | 0.054 | 0.012 | 0.251 | 0.030 | 0.014 |
| 440400 | 0.013 | 0.014 | 0.021 | 0.043 | 0.063 | 0.212 | 0.212 | 0.181 | 0.014 | 0.169 | 0.040 | 0.018 |
| 440500 | 0.016 | 0.016 | 0.019 | 0.050 | 0.066 | 0.229 | 0.225 | 0.161 | 0.012 | 0.156 | 0.035 | 0.016 |
| 440600 | 0.014 | 0.019 | 0.019 | 0.042 | 0.054 | 0.252 | 0.212 | 0.157 | 0.018 | 0.171 | 0.032 | 0.011 |
| 440700 | 0.015 | 0.015 | 0.020 | 0.042 | 0.060 | 0.234 | 0.220 | 0.165 | 0.014 | 0.160 | 0.039 | 0.016 |
| 440800 | 0.016 | 0.015 | 0.022 | 0.048 | 0.068 | 0.216 | 0.229 | 0.163 | 0.013 | 0.151 | 0.042 | 0.017 |
| 440900 | 0.015 | 0.015 | 0.022 | 0.049 | 0.066 | 0.217 | 0.223 | 0.164 | 0.015 | 0.149 | 0.046 | 0.019 |
| 441200 | 0.016 | 0.017 | 0.021 | 0.045 | 0.070 | 0.221 | 0.225 | 0.159 | 0.015 | 0.154 | 0.040 | 0.018 |
| 441300 | 0.016 | 0.019 | 0.019 | 0.041 | 0.065 | 0.235 | 0.217 | 0.164 | 0.013 | 0.156 | 0.037 | 0.017 |
| 441400 | 0.016 | 0.014 | 0.022 | 0.047 | 0.067 | 0.225 | 0.220 | 0.162 | 0.015 | 0.146 | 0.043 | 0.022 |
| 441500 | 0.016 | 0.019 | 0.021 | 0.042 | 0.071 | 0.221 | 0.215 | 0.162 | 0.018 | 0.141 | 0.050 | 0.025 |
| 441600 | 0.017 | 0.020 | 0.022 | 0.045 | 0.069 | 0.222 | 0.216 | 0.162 | 0.016 | 0.142 | 0.049 | 0.021 |
| 441700 | 0.015 | 0.020 | 0.022 | 0.048 | 0.068 | 0.226 | 0.217 | 0.162 | 0.016 | 0.147 | 0.044 | 0.016 |
| 441800 | 0.014 | 0.020 | 0.021 | 0.044 | 0.069 | 0.226 | 0.214 | 0.163 | 0.019 | 0.149 | 0.044 | 0.017 |
| 441900 | 0.014 | 0.030 | 0.026 | 0.051 | 0.031 | 0.293 | 0.082 | 0.173 | 0.012 | 0.232 | 0.040 | 0.015 |
| 442000 | 0.011 | 0.017 | 0.021 | 0.039 | 0.055 | 0.248 | 0.184 | 0.170 | 0.019 | 0.171 | 0.047 | 0.018 |
| 445100 | 0.016 | 0.017 | 0.020 | 0.046 | 0.071 | 0.225 | 0.217 | 0.164 | 0.015 | 0.148 | 0.046 | 0.015 |
| 445200 | 0.018 | 0.019 | 0.021 | 0.046 | 0.071 | 0.221 | 0.222 | 0.161 | 0.015 | 0.145 | 0.043 | 0.018 |
| 445300 | 0.017 | 0.019 | 0.022 | 0.044 | 0.066 | 0.220 | 0.213 | 0.160 | 0.019 | 0.145 | 0.055 | 0.022 |
| 450100 | 0.015 | 0.020 | 0.021 | 0.046 | 0.064 | 0.177 | 0.257 | 0.163 | 0.013 | 0.169 | 0.038 | 0.017 |
| 450200 | 0.015 | 0.015 | 0.020 | 0.045 | 0.061 | 0.222 | 0.228 | 0.166 | 0.016 | 0.155 | 0.039 | 0.017 |
| 450300 | 0.016 | 0.016 | 0.020 | 0.047 | 0.067 | 0.208 | 0.232 | 0.164 | 0.018 | 0.148 | 0.043 | 0.020 |
| 450400 | 0.017 | 0.018 | 0.022 | 0.047 | 0.065 | 0.220 | 0.219 | 0.162 | 0.016 | 0.145 | 0.047 | 0.021 |
| 450500 | 0.017 | 0.021 | 0.020 | 0.045 | 0.066 | 0.218 | 0.222 | 0.165 | 0.015 | 0.145 | 0.045 | 0.020 |
| 450600 | 0.016 | 0.024 | 0.022 | 0.048 | 0.066 | 0.223 | 0.214 | 0.161 | 0.018 | 0.139 | 0.048 | 0.022 |
| 450700 | 0.017 | 0.023 | 0.022 | 0.049 | 0.059 | 0.218 | 0.222 | 0.163 | 0.015 | 0.148 | 0.044 | 0.021 |
| 450800 | 0.016 | 0.019 | 0.023 | 0.048 | 0.068 | 0.216 | 0.219 | 0.161 | 0.019 | 0.147 | 0.044 | 0.021 |
| 450900 | 0.017 | 0.019 | 0.022 | 0.046 | 0.067 | 0.218 | 0.225 | 0.161 | 0.017 | 0.145 | 0.042 | 0.021 |
| 451000 | 0.018 | 0.018 | 0.023 | 0.047 | 0.064 | 0.213 | 0.220 | 0.162 | 0.017 | 0.146 | 0.048 | 0.023 |
| 451100 | 0.017 | 0.020 | 0.022 | 0.047 | 0.068 | 0.218 | 0.216 | 0.161 | 0.018 | 0.141 | 0.049 | 0.022 |
| 451200 | 0.017 | 0.022 | 0.023 | 0.047 | 0.063 | 0.215 | 0.217 | 0.160 | 0.018 | 0.143 | 0.048 | 0.026 |
| 451300 | 0.017 | 0.021 | 0.023 | 0.047 | 0.066 | 0.218 | 0.216 | 0.160 | 0.020 | 0.145 | 0.046 | 0.021 |
| 451400 | 0.018 | 0.022 | 0.022 | 0.048 | 0.069 | 0.206 | 0.215 | 0.161 | 0.018 | 0.145 | 0.051 | 0.025 |
| 460100 | 0.012 | 0.026 | 0.023 | 0.044 | 0.053 | 0.207 | 0.244 | 0.171 | 0.012 | 0.146 | 0.045 | 0.018 |
| 460200 | 0.014 | 0.024 | 0.023 | 0.053 | 0.072 | 0.221 | 0.238 | 0.181 | 0.015 | 0.075 | 0.058 | 0.025 |
| 500000 | 0.023 | 0.028 | 0.026 | 0.068 | 0.067 | 0.158 | 0.171 | 0.164 | 0.018 | 0.241 | 0.026 | 0.012 |
| 510100 | 0.019 | 0.008 | 0.023 | 0.055 | 0.063 | 0.169 | 0.204 | 0.163 | 0.023 | 0.226 | 0.032 | 0.016 |
| 510300 | 0.016 | 0.011 | 0.022 | 0.044 | 0.067 | 0.221 | 0.220 | 0.162 | 0.022 | 0.145 | 0.049 | 0.020 |
| 510400 | 0.015 | 0.025 | 0.024 | 0.047 | 0.058 | 0.230 | 0.200 | 0.167 | 0.013 | 0.154 | 0.050 | 0.017 |
| 510500 | 0.018 | 0.019 | 0.022 | 0.046 | 0.068 | 0.216 | 0.222 | 0.162 | 0.019 | 0.152 | 0.039 | 0.018 |
| 510600 | 0.016 | 0.019 | 0.021 | 0.045 | 0.067 | 0.215 | 0.221 | 0.164 | 0.019 | 0.151 | 0.044 | 0.019 |
| 510700 | 0.016 | 0.019 | 0.021 | 0.041 | 0.066 | 0.213 | 0.229 | 0.166 | 0.016 | 0.155 | 0.039 | 0.019 |
| 510800 | 0.018 | 0.019 | 0.022 | 0.047 | 0.065 | 0.225 | 0.218 | 0.162 | 0.015 | 0.143 | 0.045 | 0.021 |
| 510900 | 0.018 | 0.022 | 0.022 | 0.048 | 0.066 | 0.223 | 0.215 | 0.161 | 0.018 | 0.142 | 0.041 | 0.023 |
| 511000 | 0.018 | 0.020 | 0.022 | 0.048 | 0.067 | 0.217 | 0.216 | 0.157 | 0.021 | 0.148 | 0.046 | 0.019 |
| 511100 | 0.016 | 0.020 | 0.021 | 0.047 | 0.065 | 0.219 | 0.217 | 0.161 | 0.020 | 0.149 | 0.044 | 0.020 |
| 511300 | 0.019 | 0.019 | 0.021 | 0.048 | 0.065 | 0.218 | 0.225 | 0.162 | 0.017 | 0.150 | 0.038 | 0.020 |
| 511400 | 0.017 | 0.019 | 0.021 | 0.045 | 0.068 | 0.220 | 0.217 | 0.161 | 0.021 | 0.147 | 0.042 | 0.021 |
| 511500 | 0.017 | 0.020 | 0.021 | 0.045 | 0.067 | 0.219 | 0.219 | 0.160 | 0.021 | 0.147 | 0.043 | 0.021 |
| 511600 | 0.017 | 0.018 | 0.021 | 0.047 | 0.070 | 0.220 | 0.217 | 0.159 | 0.019 | 0.143 | 0.046 | 0.024 |
| 511700 | 0.018 | 0.020 | 0.021 | 0.047 | 0.068 | 0.216 | 0.218 | 0.159 | 0.019 | 0.146 | 0.048 | 0.021 |
| 511800 | 0.017 | 0.020 | 0.023 | 0.051 | 0.063 | 0.210 | 0.218 | 0.164 | 0.017 | 0.142 | 0.051 | 0.023 |
| 511900 | 0.019 | 0.023 | 0.022 | 0.049 | 0.066 | 0.221 | 0.216 | 0.159 | 0.018 | 0.137 | 0.049 | 0.021 |
| 512000 | 0.017 | 0.020 | 0.021 | 0.049 | 0.063 | 0.222 | 0.217 | 0.160 | 0.021 | 0.141 | 0.045 | 0.022 |
| 520100 | 0.016 | 0.022 | 0.021 | 0.046 | 0.066 | 0.167 | 0.245 | 0.173 | 0.016 | 0.171 | 0.041 | 0.017 |
| 520200 | 0.016 | 0.015 | 0.022 | 0.047 | 0.061 | 0.220 | 0.220 | 0.163 | 0.017 | 0.146 | 0.050 | 0.022 |
| 520300 | 0.016 | 0.018 | 0.022 | 0.046 | 0.066 | 0.213 | 0.227 | 0.162 | 0.017 | 0.149 | 0.044 | 0.018 |
| 520400 | 0.016 | 0.018 | 0.023 | 0.046 | 0.070 | 0.215 | 0.218 | 0.162 | 0.016 | 0.147 | 0.047 | 0.021 |
| 530100 | 0.014 | 0.023 | 0.022 | 0.045 | 0.061 | 0.143 | 0.264 | 0.176 | 0.012 | 0.186 | 0.036 | 0.018 |
| 530300 | 0.017 | 0.013 | 0.023 | 0.045 | 0.068 | 0.216 | 0.223 | 0.162 | 0.015 | 0.151 | 0.044 | 0.023 |
| 530400 | 0.015 | 0.019 | 0.022 | 0.044 | 0.064 | 0.225 | 0.220 | 0.163 | 0.015 | 0.142 | 0.048 | 0.022 |
| 530500 | 0.017 | 0.020 | 0.023 | 0.048 | 0.066 | 0.215 | 0.215 | 0.159 | 0.017 | 0.143 | 0.051 | 0.025 |
| 530600 | 0.019 | 0.019 | 0.024 | 0.048 | 0.063 | 0.210 | 0.216 | 0.159 | 0.019 | 0.147 | 0.050 | 0.026 |
| 530700 | 0.016 | 0.022 | 0.023 | 0.048 | 0.071 | 0.217 | 0.219 | 0.159 | 0.012 | 0.135 | 0.052 | 0.027 |
| 530800 | 0.018 | 0.024 | 0.024 | 0.045 | 0.068 | 0.220 | 0.216 | 0.160 | 0.016 | 0.136 | 0.051 | 0.023 |
| 530900 | 0.018 | 0.022 | 0.024 | 0.048 | 0.068 | 0.216 | 0.212 | 0.158 | 0.016 | 0.140 | 0.053 | 0.026 |
| 610100 | 0.015 | 0.024 | 0.021 | 0.045 | 0.072 | 0.108 | 0.283 | 0.166 | 0.021 | 0.198 | 0.031 | 0.017 |
| 610200 | 0.017 | 0.018 | 0.022 | 0.050 | 0.064 | 0.226 | 0.212 | 0.160 | 0.017 | 0.142 | 0.051 | 0.022 |
| 610300 | 0.017 | 0.023 | 0.020 | 0.045 | 0.064 | 0.223 | 0.219 | 0.161 | 0.015 | 0.149 | 0.042 | 0.021 |
| 610400 | 0.018 | 0.017 | 0.020 | 0.047 | 0.066 | 0.204 | 0.225 | 0.162 | 0.021 | 0.153 | 0.047 | 0.020 |
| 610500 | 0.018 | 0.017 | 0.021 | 0.047 | 0.063 | 0.222 | 0.216 | 0.159 | 0.024 | 0.150 | 0.042 | 0.021 |
| 610600 | 0.016 | 0.019 | 0.022 | 0.048 | 0.064 | 0.221 | 0.216 | 0.155 | 0.018 | 0.146 | 0.055 | 0.021 |
| 610700 | 0.016 | 0.020 | 0.021 | 0.047 | 0.058 | 0.219 | 0.221 | 0.162 | 0.017 | 0.147 | 0.049 | 0.023 |
| 610800 | 0.015 | 0.019 | 0.021 | 0.046 | 0.064 | 0.223 | 0.221 | 0.159 | 0.017 | 0.148 | 0.043 | 0.021 |
| 610900 | 0.018 | 0.019 | 0.022 | 0.049 | 0.065 | 0.219 | 0.219 | 0.161 | 0.016 | 0.140 | 0.048 | 0.024 |
| 611000 | 0.018 | 0.021 | 0.022 | 0.048 | 0.067 | 0.214 | 0.212 | 0.159 | 0.020 | 0.141 | 0.053 | 0.025 |
| 620100 | 0.014 | 0.022 | 0.024 | 0.042 | 0.062 | 0.167 | 0.249 | 0.170 | 0.019 | 0.171 | 0.045 | 0.014 |
| 620200 | 0.014 | 0.026 | 0.029 | 0.056 | 0.062 | 0.252 | 0.226 | 0.173 | 0.017 | 0.060 | 0.060 | 0.024 |
| 620300 | 0.015 | 0.030 | 0.025 | 0.050 | 0.063 | 0.226 | 0.212 | 0.160 | 0.017 | 0.121 | 0.058 | 0.024 |
| 620400 | 0.016 | 0.024 | 0.024 | 0.048 | 0.069 | 0.220 | 0.213 | 0.158 | 0.018 | 0.141 | 0.050 | 0.019 |
| 620500 | 0.018 | 0.020 | 0.026 | 0.048 | 0.069 | 0.212 | 0.219 | 0.159 | 0.016 | 0.146 | 0.047 | 0.022 |
| 620600 | 0.018 | 0.022 | 0.025 | 0.045 | 0.055 | 0.219 | 0.218 | 0.161 | 0.020 | 0.139 | 0.054 | 0.024 |
| 620700 | 0.016 | 0.024 | 0.025 | 0.047 | 0.066 | 0.219 | 0.217 | 0.158 | 0.017 | 0.134 | 0.053 | 0.024 |
| 620800 | 0.017 | 0.023 | 0.024 | 0.048 | 0.065 | 0.217 | 0.215 | 0.158 | 0.019 | 0.139 | 0.049 | 0.026 |
| 620900 | 0.014 | 0.024 | 0.022 | 0.048 | 0.063 | 0.226 | 0.218 | 0.158 | 0.017 | 0.134 | 0.052 | 0.025 |
| 621000 | 0.017 | 0.022 | 0.023 | 0.048 | 0.067 | 0.214 | 0.215 | 0.158 | 0.020 | 0.139 | 0.052 | 0.025 |
| 621100 | 0.018 | 0.021 | 0.025 | 0.046 | 0.065 | 0.214 | 0.211 | 0.156 | 0.020 | 0.141 | 0.054 | 0.029 |
| 621200 | 0.017 | 0.022 | 0.024 | 0.050 | 0.060 | 0.212 | 0.209 | 0.156 | 0.025 | 0.137 | 0.060 | 0.028 |
| 630100 | 0.014 | 0.024 | 0.024 | 0.044 | 0.053 | 0.210 | 0.235 | 0.166 | 0.018 | 0.152 | 0.045 | 0.016 |
| 640100 | 0.014 | 0.021 | 0.023 | 0.044 | 0.059 | 0.198 | 0.235 | 0.162 | 0.017 | 0.162 | 0.049 | 0.016 |
| 640200 | 0.015 | 0.022 | 0.025 | 0.048 | 0.065 | 0.228 | 0.211 | 0.162 | 0.017 | 0.134 | 0.053 | 0.020 |
| 640300 | 0.017 | 0.025 | 0.023 | 0.046 | 0.061 | 0.222 | 0.214 | 0.160 | 0.015 | 0.140 | 0.056 | 0.021 |
| 640400 | 0.016 | 0.024 | 0.028 | 0.048 | 0.063 | 0.217 | 0.211 | 0.159 | 0.018 | 0.140 | 0.050 | 0.026 |
| 640500 | 0.016 | 0.026 | 0.026 | 0.046 | 0.069 | 0.222 | 0.209 | 0.158 | 0.018 | 0.136 | 0.055 | 0.019 |
| 650100 | 0.012 | 0.029 | 0.023 | 0.043 | 0.049 | 0.186 | 0.243 | 0.161 | 0.018 | 0.178 | 0.043 | 0.014 |
| 650200 | 0.014 | 0.025 | 0.021 | 0.053 | 0.054 | 0.247 | 0.204 | 0.157 | 0.013 | 0.137 | 0.054 | 0.020 |
